# Cardiovascular and haematological events post COVID‐19 vaccination: A systematic review

**DOI:** 10.1111/jcmm.17137

**Published:** 2021-12-29

**Authors:** Dana Al‐Ali, Abdallah Elshafeey, Malik Mushannen, Hussam Kawas, Ameena Shafiq, Narjis Mhaimeed, Omar Mhaimeed, Nada Mhaimeed, Rached Zeghlache, Mohammad Salameh, Pradipta Paul, Moayad Homssi, Ibrahim Mohammed, Adeeb Narangoli, Lina Yagan, Bushra Khanjar, Sa’ad Laws, Mohamed B. Elshazly, Dalia Zakaria

**Affiliations:** ^1^ Weill Cornell Medicine Qatar Doha Qatar; ^2^ Department of Cardiovascular Medicine Cleveland Clinic Cleveland Ohio USA

**Keywords:** COVID‐19, haemorrhage, myocardial infraction, myocarditis, myopericarditis, pericarditis, thrombocytopenia, thrombosis, vaccine

## Abstract

Since COVID‐19 took a strong hold around the globe causing considerable morbidity and mortality, a lot of effort was dedicated to manufacturing effective vaccines against SARS‐CoV‐2. Many questions have since been raised surrounding the safety of the vaccines, and a lot of media attention to certain side effects. This caused a state of vaccine hesitancy that may prove problematic in the global effort to control the virus. This review was undertaken with the aim of putting together all the reported cardiovascular and haematological events post COVID‐19 vaccination in published literature and to suggest possible mechanisms to explain these rare phenomena.

## 1 INTRODUCTION

In 2019, the world first witnessed the emergence of the novel coronavirus, now known as SARS‐CoV‐2, in Wuhan, China. It was the cause of severe respiratory disease, spreading across the world at an unprecedented speed, leading to global alarm and declaration of coronavirus disease 2019 (COVID‐19) as a pandemic. As of June 15^th^, 2021, there have been 1.76 billion reported COVID‐19 cases worldwide and more than 38 million deaths according to the World Health Organization (WHO).^1^ The enormous effect of COVID‐19 on the health systems and economies of countries all over the world has driven the scientific community to come together to develop numerous diagnostic and therapeutic modalities to help curb the spread of the pandemic. Hastened manufacture and rollout of newly developed vaccines has led to the biggest vaccination campaign in history, but has left many questioning whether safety has been compromised. Furthermore, emerging reports of adverse effects of many of these vaccines have contributed to vaccine hesitancy in the community, thus becoming a hurdle in controlling the spread of COVID‐19.

More than 200 COVID‐19 vaccines are under development, with over 60 being tested in clinical trials.^2^ The first rollout of COVID‐19 vaccinations began in December 2020. The most prominent of which are the Pfizer‐BioNTech, Moderna, Oxford–AstraZeneca, Johnson and Johnson Janssen (J&J) and the CoronaVac, Sinovac Life Sciences vaccines. To this date, more than 2.39 billion doses of vaccines have been administered across 178 countries at a rate of 36.3 million doses per day.^3^ It is estimated that around 15%–16% of the global population has been vaccinated.^3^

Many adverse events were reported post COVID‐19 vaccinations. The U.S. FDA Center for Biologics Evaluation and Research published a protocol on background Rates of Adverse Events of Special Interest (AESIs) for COVID‐19 Vaccine Safety Monitoring. Acute myocardial infarction, anaphylaxis, appendicitis, Bell's palsy, deep vein thrombosis, disseminated intravascular coagulation, encephalomyelitis, Guillain–Barre syndrome, haemorrhagic and non‐haemorrhagic stroke, immune thrombocytopenia, myocarditis/pericarditis, narcolepsy, pulmonary embolism and transverse myelitis were listed as the ASEIs of the outcome of general population.^4^

Understanding mechanisms of vaccine‐induced cardiovascular (CV) complications will help in developing vaccines with a stronger safety profile. Several types of events, including thrombotic complications, have been reported in a small number of individuals who have received the COVID‐19 vaccines. Unfortunately, such rare reports created some doubts and hesitancy about the COVID‐19 vaccines and the withdrawal of certain vaccines in some countries. It is, therefore, essential to continue monitoring the risk of rare adverse events that are not detected during the clinical trials either due to the rarity of the events or due to the long time for the onset. This review compiled all published data about the CV and haematological complications which have been reported post COVID‐19 vaccination in an attempt to reflect the true picture about the occurrence of such rare events.

## 2 METHODS

The preferred reporting items for systematic reviews and metanalysis (PRISMA) statement was used to develop the protocol of this systematic review.^5^

### 2.1 Eligibility criteria

We conducted a comprehensive literature search of clinical studies that reported any cardiovascular or haematological events post COVID‐19 vaccination. No restrictions were made about country, age or gender. Any articles that did not have any primary data, such as review articles, were excluded from the study. During the full‐text screening, only studies that specified the type of COVID‐19 vaccine after which the event appeared were selected.

### 2.2 Information sources and search strategy

We conducted a comprehensive search that prioritized sensitivity for comprehensiveness to target any studies about vaccines against COVID‐19. Appendix S1 includes the details of the databases and the search strategy for each database.

### 2.3 Study selection and data collection

During the screening phase, the studies reporting any CV or haematological events post COVID‐19 vaccination were selected. No restrictions were made about country, age or gender. Any duplicated articles were removed, and reviews or any articles that did not include primary data were excluded from the study. Studies that were not in English or those that did not specify the type of COVID‐19 vaccine were excluded. Title and abstract as well as full‐text screening were conducted by two different reviewers for each study using Covidence, and disagreements were resolved by consensus. Demographic and clinical data of patients reported in each study (whenever data were available) were extracted independently by two different reviewers using Covidence, and disagreements were resolved by consensus. Data were extracted from each study by two different reviewers.

### 2.4 Data items

Out of the selected studies, we collected the epidemiological and clinical data, including age, sex, comorbidities, treatments and outcomes. Continuous variables were expressed as mean ± standard deviation or range of results. Categorical variables were expressed as percentages.

### 2.5 Data analysis

CV and haematological events were classified into four major categories: cardiac injury (CI), thrombosis, thrombocytopenia (TP) and hemorrhage. Several cases had multiple events under different categories or within the same category. For this reason, two types of analyses were conducted: the number of cases who suffered from any type of CV and haematological events post COVID‐19 vaccination and the number of events under each category. Appendix S2 includes the details of data analysis.

## 3 RESULTS

**FIGURE 1:**
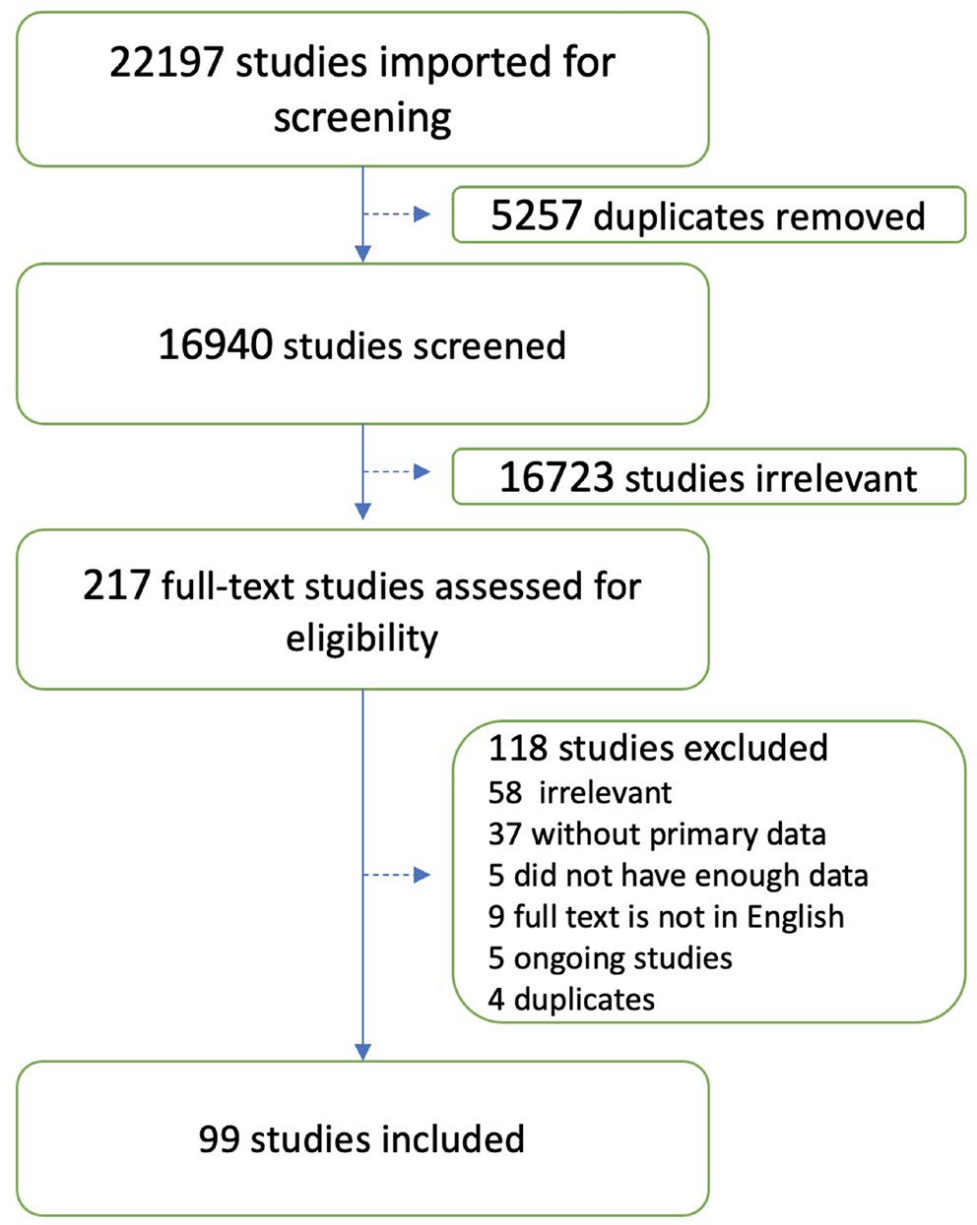
Screening and study selection protocol

Results of search and screening are summarized in Figure 1. The flow diagram shows the details of our protocol. After removing the duplicates, 16,940 studies were screened of which 217 were selected for full‐text screening. Only 99 studies were eligible to be included in this review. The excluded studies included 58 studies irrelevant to the data we were looking for, five did not have enough data, 37 had no primary data, nine were not in English, five were ongoing studies and four were duplicates.

### 3.1 Types of studies and demographic data

Tables [Link], [Link], [Link], [Link], [Link] summarize the types of studies and demographic data of the included patients who suffered from CV and haematological events following receiving Pfizer, Moderna, AstraZeneca, J&J and CoronaVac, respectively.^6^, ^7^, ^8^, ^9^, ^10^, ^11^, ^12^, ^13^, ^14^, ^15^, ^16^, ^17^, ^18^, ^19^, ^20^, ^21^, ^22^, ^23^, ^24^, ^25^, ^26^, ^27^, ^28^, ^29^, ^30^, ^31^, ^32^, ^33^, ^34^, ^35^, ^36^, ^37^, ^38^, ^39^, ^40^, ^41^, ^42^, ^43^, ^44^, ^45^, ^46^, ^47^, ^48^, ^49^, ^50^, ^51^, ^52^, ^53^, ^54^, ^55^, ^56^, ^57^, ^58^, ^59^, ^60^, ^61^, ^62^, ^63^, ^64^, ^65^, ^66^, ^67^, ^68^, ^69^, ^70^, ^71^, ^72^, ^73^, ^74^, ^75^, ^76^, ^77^, ^78^, ^79^, ^80^, ^81^, ^82^, ^83^, ^84^, ^85^, ^86^, ^87^, ^88^, ^89^, ^90^, ^91^, ^92^, ^93^, ^94^, ^95^, ^96^, ^97^, ^98^, ^99^, ^100^, ^101^, ^102^, ^103^ The included 99 studies were 52 case reports, 41 case series and two retrospective cohort studies and one retrospective descriptive study and two observational studies in Australia, Austria, Belgium, Canada, China, Denmark, France, Germany, Greece, India, Ireland, Israel, Italy, Japan, Malaysia, Mexico, Norway, Oman, Poland, Portugal, Qatar, Saudi Arabia, Singapore, Spain, Switzerland, Turkey, UK, USA and 1 multinational study. As some studies used the same databases as the source of their data, it was important to remove any duplicates. For example, Welsh et al.^22^ (Tables [Link], [Link]) used the Vaccine Adverse Event Reporting System (VAERS) and Lee et al.^21^ (Tables [Link], [Link]) used data available from the CDC, FDA, VAERS, published reports and *via* direct communication with patients and treating providers. The published reports used by Welsh et al. included two of our included studies which are by Toom et al.^53^ (Table S3) and Tarawneh et al.^43^ (Table S2) As these studies reported the individual details of each patient, it was possible to compare and combine the duplicates to avoid over counting the cases/events. Similarly, the studies conducted by Pawlowski et al.^24^ (Table S2) and Tobaiqy et al.^61^ (Table S4), who reported cases from the Mayo clinic or Eudra Vigilance (EV) database, respectively, reported a small number of cases with the individual demographic and clinical data for each patient and no duplication was detected. However, Smadja et al.^104^ obtained their data from the World Health Organization Global Database for Individual Case Safety Reports (VigiBase) and reported a large number of cases without reporting the details of each patient. It was, therefore, decided to separate the data collected from the study conducted by Smadja et al. to avoid any possibility of duplication. Furthermore, Pottegård et al.^58^ only reported the number and types of events post COVID‐19 vaccination, but not the total number of cases. As some cases had multiple events post COVID‐19 vaccination, the total number of cases, sex and age groups of the patients reported in this study was not compiled with the other included studies. However, any data concerning the number and types of events were compiled from all the included studies except those by Smadja et al.

**FIGURE 2:**
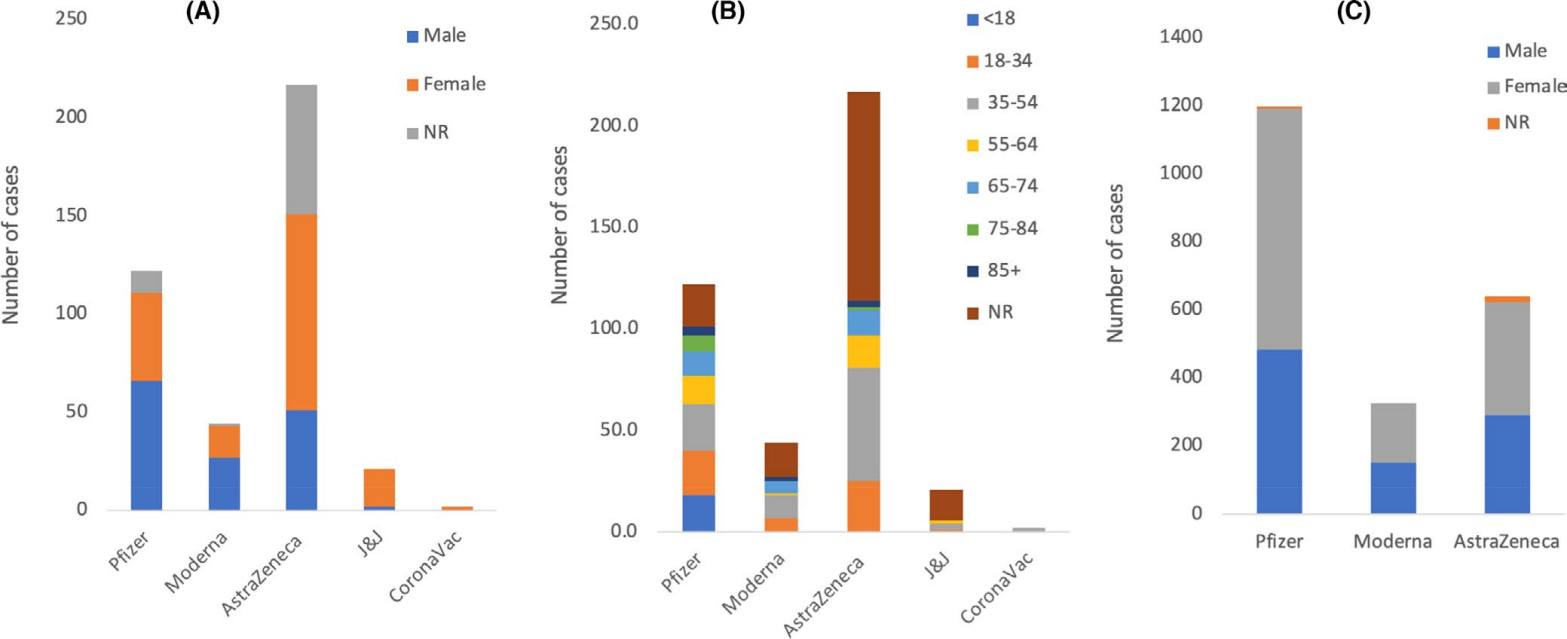
Total number of cases who experienced CV and/or haematological events following COVID‐19 vaccination and their age and gender in the included studies excluding Pottegård et al. (A) Total of 122 (54.1% M, 36.9% F and 9.0% NR), 44 (61.4% M, 36.4% F and 2.3% NR), 217 (23.5% M, 46.1% F and 30.4% NR), 21 (9.5% M and 90.5% F) and 2 (100.0% F) individuals who received Pfizer, Moderna, AstraZeneca, J&J or CoronaVac, respectively, experienced CV and haematological events. (B) Age ranges of the individuals who were diagnosed with CV and/or haematological events post COVID‐19 vaccination. The exact age was not reported (NR) for 21 cases who received Pfizer vaccine seven of which were reported as 20–51, 8 were 20–81, 1 was 31–82 years and 3 described as elderly. Moderna had 17 cases with the exact age NR of which 16 were reported as 20–51 years. Similarly, the exact age was NR for 103 individuals who received AstraZeneca vaccine 21 of which were reported as 18–64, 4 as 65–85, 19 as 22–49, 9 as 25–48, 37 as 20–89, 8 as 31–81, 8 as 21–69 and 4 as 24–53 years. Pottegard et al. reported the age range separately as 32–55. J&J had 15 cases with the exact age NR, 8 of which were reported as 18–39, 4 as ≥40, 2 as 30–39 and 1 as 50–59 years. Surprisingly, the age group of 35–54 seems to be the most affected in all the five vaccines. (C) Number of thrombotic events reported to the Vigibase database between December 13^th^ 2020 to March 16^th^ 2021 as reported by Smadja et al. Out of 361734967 receiving Pfizer, Moderna or AstraZeneca vaccines, 2161 had thrombotic events of which 1197 received Pfizer (59.1% F, 40.4 M and 0.5% NR), 325 received Moderna (53.2% F and 46.8 M) and 639 received AstraZeneca (52% F, 45.5% M and 2.5 NR). In this study, the median age was 76, 72 and 67 for Pfizer, Moderna and AstraZeneca, respectively

The studies (except Smadja et al. and Pottegård et al.) reported a total of 406 patients who received COVID‐19 vaccines and experienced one or more of the CV and/or haematological events post vaccination. The included studies reported these types of events following Pfizer, Moderna, AstraZeneca, J&J and CoronaVac vaccination. Among the 406 reported individuals, 122 (66M, 45F and 11NR) received Pfizer, 44 (27 M, 16 F and 1 NR) received Moderna, 217 (51M, 100F, and 66NR) received AstraZeneca, 21 (2 M and 19 F) received J&J and two (all females) received CoronaVac vaccination. In general, 44.8% of the total patients who received one of the five different vaccines were females. As shown in Figure 2A, there was no obvious trend in terms of gender. On the other hand, Figure 2B highlights that the age group 35–54 is the most affected in all five vaccines.

Smadja et al. reported 2161 cases in total of thrombotic events following COVID‐19 vaccination of which 1197 received Pfizer (708 F, 483 M, 6 NR), 325 received Moderna (173F, 152 M) and 639 received AstraZeneca (332 F, 291 M, 16 NR). The median and age range of patients was 76 (19–102) for Pfizer, 72 (19–102) for Moderna and 67 (19–99) for AstraZeneca (Figure 2C).

### 3.2 Clinical data

Tables [Link], [Link], [Link], [Link], [Link] summarize the clinical features of the COVID‐19–vaccinated individuals who suffered from CV and/or haematological events after vaccination, including clinical progression, outcomes, treatments and laboratory markers.

**FIGURE 3:**
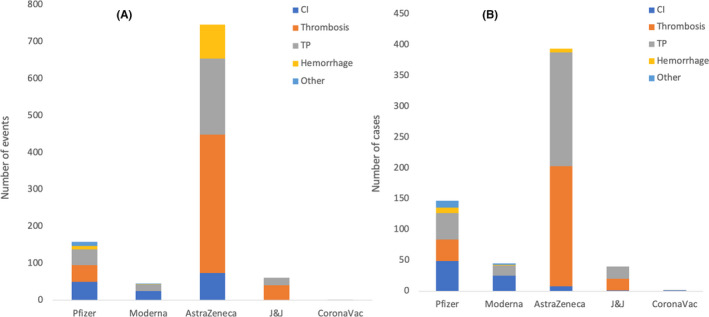
Total number and types of cardiovascular (CV) and haematological events reported following COVID‐19 vaccination in 98 included studies, excluding that by Smadja et al. The results of the study by Pottegård et al. are included where events are reported and excluded from cases. A total of 158 CV and haematological events were reported in 122 individuals who received Pfizer vaccine, some of whom suffered from multiple events (50 cardiac injury (CI), 45 thrombosis, 43 thrombocytopenia (TP), 9 haemorrhage and 11 others, including 8 stage 2 hypertension (HTN) and 3 microangiopathy). A total of 45 cases experienced CV and haematological events following receiving Moderna vaccine (25 CI and 17 TP without thrombosis, 1 hemorrhage and 2 others, including 1 stage 2 HTN and 1 hypertensive crisis). Two hundred and seventeen individuals who received AstraZeneca vaccine experienced 747 CV and haematological events as some of them suffered from multiple events (74 cardiac problems, 375 thrombosis, 206 TP and 92 haemorrhage). Sixty‐one events were experienced by 21 individuals who received the J&J vaccine (1 cardiac, 40 thrombosis and 20 TP). A total of two cases experienced CV and haematological events following receiving CoronaVac vaccine (1 Kounis Syndrome, Type I variant and 1 haemophagocytic lymphohistiocytosis). As several individuals experienced multiple events, (A) illustrates the total number of events reported under each category while (B) illustrates the number of individuals who experienced each type of event, regardless of the number of events that each individual had under the same category. However, the categories may overlap as some cases had more than one event from the other major categories presented in the figures

Figure 3A illustrates the total number and types of CV and haematological events reported following COVID‐19 vaccination by the included studies (except Smadja et al.). The results of Pottegård et al. are included where events are reported and excluded from the total number of cases. A total of 158 CV and haematological events were reported in 122 individuals who received the Pfizer vaccine (50 CI, 45 thrombosis, 43 TP, nine hemorrhage and 11 others including eight stage 2 hypertension (HTN) and three microangiopathy). A total of 45 CV and haematological events were experienced following receiving the Moderna vaccine (25 CI and 17 TP without thrombosis, one haemorrhage and two others including one stage 2 HTN and one hypertensive crisis). Two hundred and seventeen individuals who received AstraZeneca vaccine experienced 747 CV and haematological events as some of them suffered from multiple events (74 cardiac problems, 375 thrombosis, 206 TP and 92 haemorrhage). Sixty‐one events were experienced by 21 individuals who received the J&J vaccine (one cardiac, 40 thrombosis and 20 TP). A total of two cases experienced CV and haematological events following receiving the CoronaVac vaccine (one Kounis Syndrome, Type I variant and 1 haemophagocytic lymphohistiocytosis). As several individuals experienced multiple events, Figure 3A illustrates the total number of events reported under each category, while Figure 3B illustrates the number of individuals who experienced each type of event regardless of the number of events that each individual had under the same category. However, the categories may overlap as some cases had more than one event from the other major categories as illustrated in Tables [Link], [Link], [Link], [Link], [Link].

Smadja et al., reported 2161 cases of thrombotic events; some were associated with MI and TP. In the studies that reported the time to onset of post‐vaccine events, the time ranges were minutes‐30 days, minutes‐23 days, 0–24 days, 5–37 days and minutes‐1 day for the Pfizer, Moderna, AstraZeneca, J&J and CoronaVac, respectively.

#### 3.2.1 Cardiac injury

**FIGURE 4:**
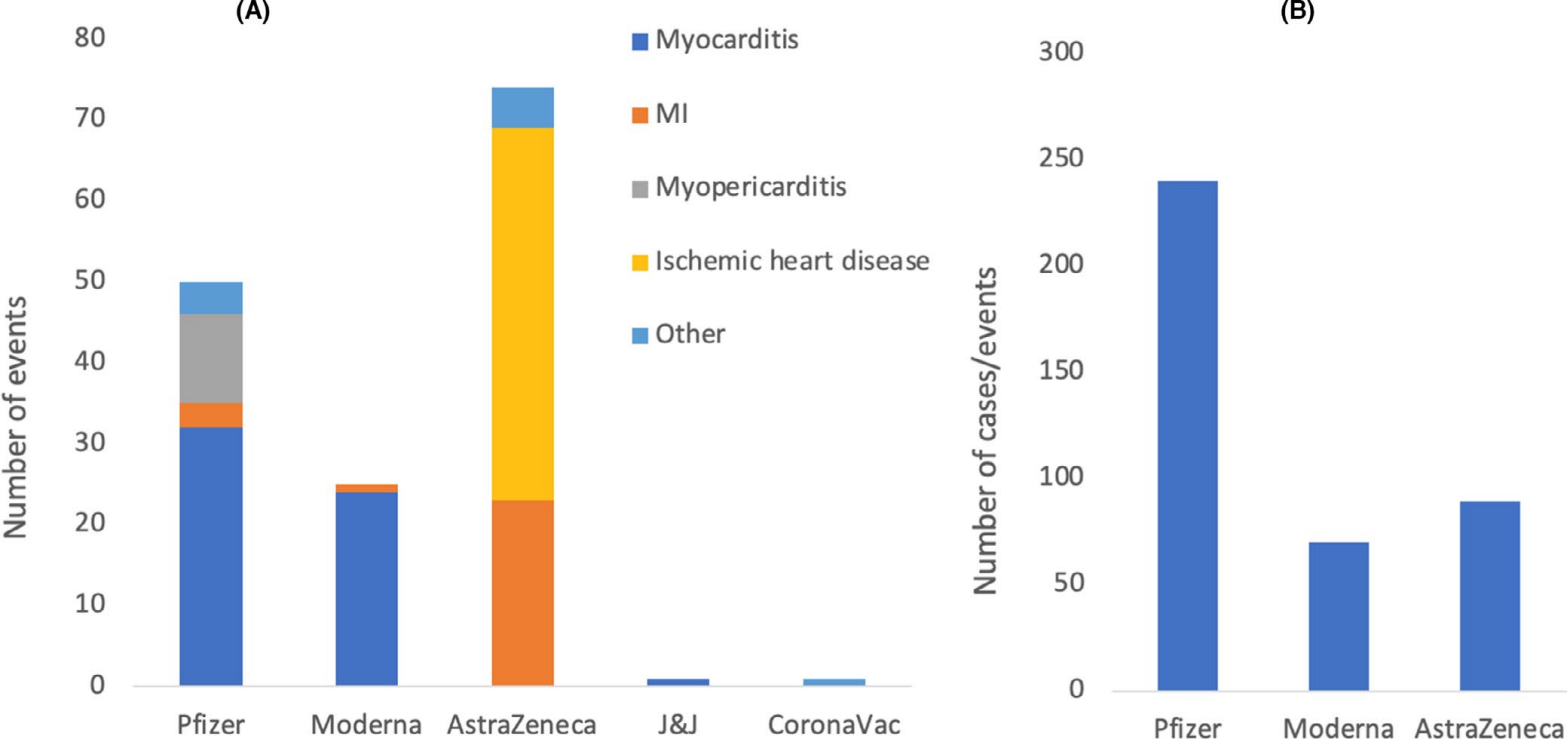
(A) Number and types of cardiac events reported post COVID‐19 vaccinations in 98 studies including those by Pottegård et al. and excluding those by Smadja et al. (reported separately). A total of 50 CI events were reported after receiving Pfizer vaccine (32 myocarditis, 3 myocardial infarction (MI), 11 myopericarditis and 4 others, including 2 pericarditis, 1 acute coronary syndrome (ACS) and 1 stress cardiomyopathy). Twenty‐five cases of cardiac events were detected in individuals post Moderna vaccination (24 myocarditis and 1 MI). Seventy‐four cardiac events were reported in individuals who received the AstraZeneca vaccine (23 MI, 46 ischemic heart disease and 5 others, including 1 heart strain, 1 cardiac arrest and 3 ACS). Only 1 case of myocarditis was reported post J&J vaccination and 1 case of Kounis syndrome type 1 variant reported after receiving the CoronaVac vaccine. (B) Number of cases of MI (associated with arterial thrombosis) in individuals who received Pfizer, Moderna and AstraZeneca vaccine as reported by Smadja et al. The cases represent 20%, 21.5% and 13.9% of the total cases of thrombosis following Pfizer, Moderna and AstraZeneca vaccines, respectively

Among the included studies, (except Smadja et al.) a total of 50 CI events were reported after receiving the Pfizer vaccine (32 myocarditis, three MI, 11 myopericarditis and four other, including two pericarditis, one ACS and one stress cardiomyopathy) (Figure 4A). Twenty‐five cases of cardiac events were detected in individuals post Moderna vaccination (24 myocarditis and one MI). Seventy‐four cardiac events were reported in individuals who received the AstraZeneca vaccine (23 MI, 46 ischemic heart disease and five others, including one heart strain, one cardiac arrest and three ACS). Only one case of myocarditis was reported post J&J vaccination and one case of Kounis syndrome type 1 variant reported after receiving the CoronaVac vaccine. Smadja et al. reported 240, 70 and 89 cases of MI associated with arterial thrombosis after receiving Pfizer, Moderna and AstraZeneca vaccines. Such cases represent 20%, 21.5% and 13.9% of the total cases of thrombosis following Pfizer, Moderna and AstraZeneca vaccines, respectively (Figure 4B).

#### 3.2.2 Haemorrhage

**FIGURE 5:**
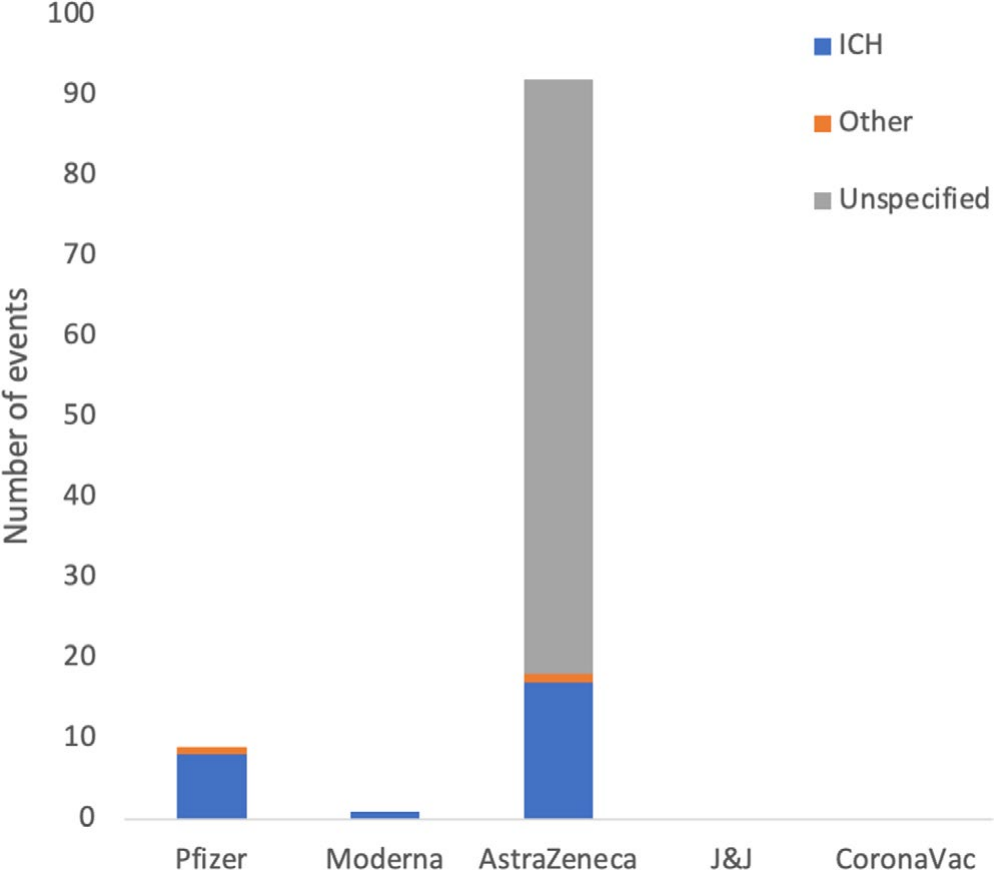
Number and types of haemorrhagic cases/events occurring not in the context of thrombocytopenia reported following COVID‐19 vaccination in 98 studies, including the studies by Pottegård et al. and excluding those by Smadja et al. A total of 9 haemorrhage cases were reported after receiving Pfizer vaccine (8 intracranial haemorrhage (ICH) and 1 case of acquired haemophilia) while one case of ICH was reported post Moderna vaccination. Additionally, 17 events of cerebral haemorrhage (4 of which were with suspected thrombocytopenia), 74 of unspecified bleeding and 1 other (uterine bleeding) were reported following AstraZeneca vaccination. No haemorrhagic events were reported after the J&J and the CoronaVac vaccine

The number and types of haemorrhagic cases/events occurring not in the context of thrombocytopenia reported following COVID‐19 vaccination are shown in Figure 5. A total of nine haemorrhage cases were reported after receiving Pfizer vaccine (eight ICH and one case of acquired haemophilia) while one case of ICH was reported post Moderna vaccination. Additionally, 17 events of cerebral hemorrhage (four of which were with suspected thrombocytopenia), 74 of unspecified bleeding and one other type of haemorrhage (uterine bleeding) were reported following AstraZeneca vaccination. No haemorrhagic events were reported after J&J and the CoronaVac vaccine.

#### 3.2.3 Thrombotic events (with or without thrombocytopenia)

**FIGURE 6:**
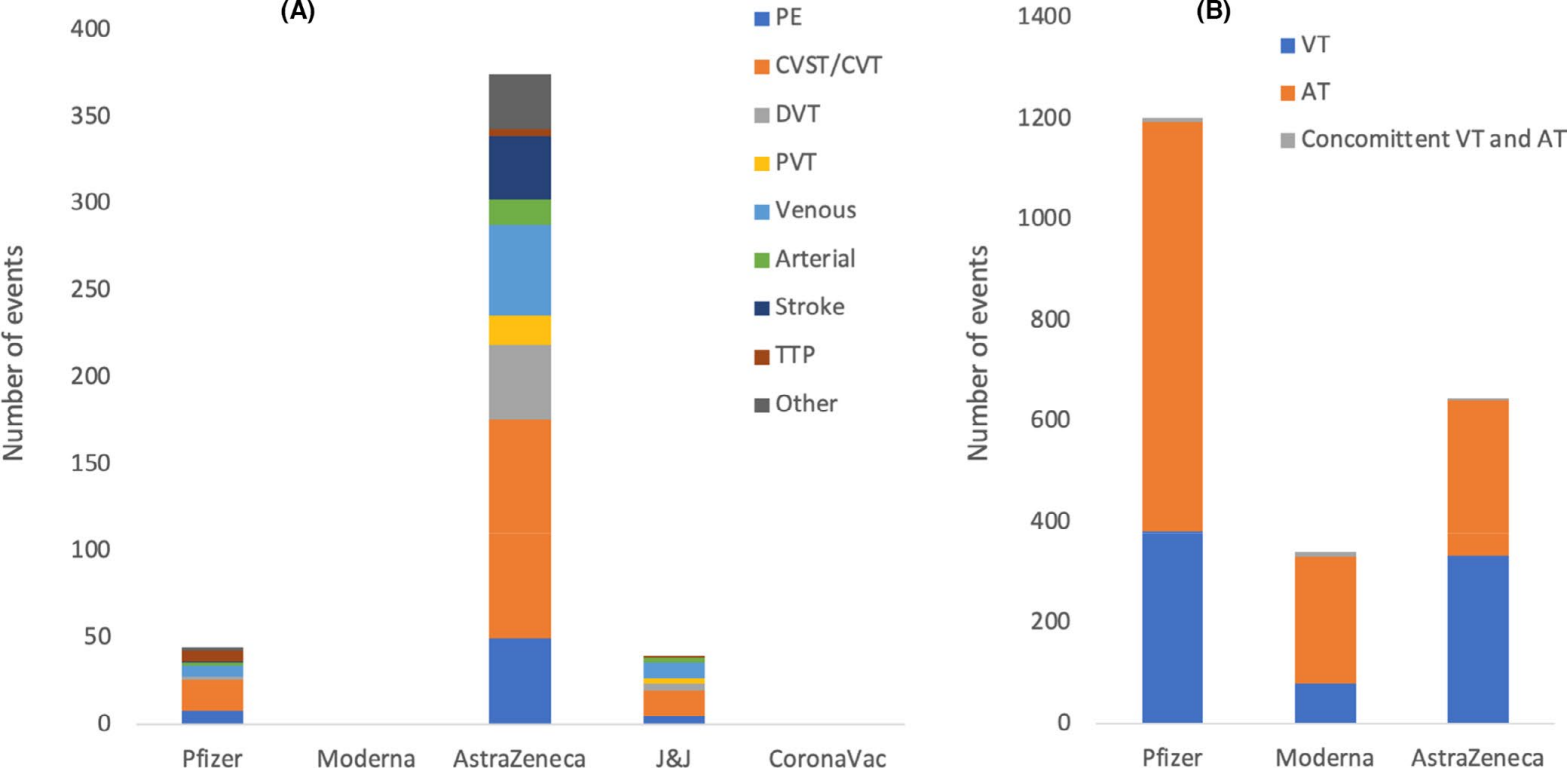
(A) Number and types of all thrombotic events with or without other events reported post COVID‐19 vaccinations by 98 studies including those by Pottegård et al. and excluding Smadja et al. (reported separately). A total of 159 cerebral venous thrombosis (CVT) events were reported post vaccination (18 Pfizer, 126 AstraZeneca and 15 J&J). Forty‐nine deep vein thrombosis events were reported post vaccination (2 Pfizer, 43 AstraZeneca and 4 J&J). A total of 63 pulmonary embolism (PE) cases were reported post vaccination (8 Pfizer, 50 AstraZeneca and 5 J&J). Portal vein thrombosis (PVT) was reported in 20 individuals (17 AstraZeneca and 3 J&J). A total of 67 venous thrombosis (6 Pfizer, 52 AstraZeneca and 9 J&J) and 20 arterial thrombosis (2 Pfizer, 15 AstraZeneca and 3 J&J) cases were reported post vaccination. Thirty‐seven stroke events (1 Pfizer and 36 AstraZeneca) and 11 thrombotic thrombocytopenic purpura (TTP) events (6 Pfizer, 4 AstraZeneca and 1 J&J) were reported post vaccination. One case of blue toes and one case of thrombophlebitis was described after receiving the Pfizer vaccine. Thirty‐two other thrombotic events were reported after receiving the AstraZeneca vaccine that include 9 organ thrombosis/infarction, 3 thrombophlebitis, 2 thrombotic microangiopathy, 1 transient ischemic attack (TIA) and 17 other unspecified thrombotic events. In general, many of the individuals who experienced cardiac or haematological complications post vaccinations had comorbidities/risk factors that may increase their chances to develop such complications. It is important to note that the number of events in this figure does not reflect the number of individuals as some of them had more than one thrombotic event. (B) Number and types of thrombotic events with or without TP as reported by Smadja et al. after receiving Pfizer (381 VT, 813 AT and 10 VT and AT), Moderna (80 VT, 253 AT and 8 VT and AT) and AstraZeneca (334 VT, 308 AT and 4 VT and AT)

Figure 6A highlights a total of 159 CVT events reported post vaccination (18 Pfizer, 126 AstraZeneca and 15 J&J). Forty‐nine DVT events were reported after COVID‐19 vaccination (two Pfizer, 43 AstraZeneca and four J&J). A total of 63 PE events were reported post vaccination (eight Pfizer, 50 AstraZeneca and five J&J). PVT was reported in 20 individuals (17 AstraZeneca and three J&J). A total of 67 venous thrombosis (six Pfizer, 52 AstraZeneca and nine J&J) and 20 arterial thrombosis (two Pfizer, 15 AstraZeneca and three J&J) events were reported post vaccination. Thirty‐seven stroke events (one Pfizer and 36 AstraZeneca) and 11 TTP events (six Pfizer, four AstraZeneca and one J&J) were reported post vaccination. One case of blue toes and one case of thrombophlebitis were described after receiving the Pfizer vaccine. Thirty‐two other thrombotic events were reported after receiving the AstraZeneca vaccine that include nine organ thrombosis/infarction, three thrombophlebitis, two thrombotic microangiopathy, one TIA and 17 other unspecified thrombotic events.

Smadja et al., reported 381 cases of venous thrombosis for Pfizer, 80 cases for Moderna and 334 cases for AstraZeneca. Arterial thrombosis was reported in 813 patients after Pfizer, in 253 patients after Moderna and in 308 patients after AstraZeneca vaccination. Furthermore, concomitant arterial and venous thrombosis was reported in 10, eight and four cases following Pfizer, Moderna or AstraZeneca vaccination, respectively (Figure 6B).

#### 3.2.4 Thrombocytopenia

**FIGURE 7:**
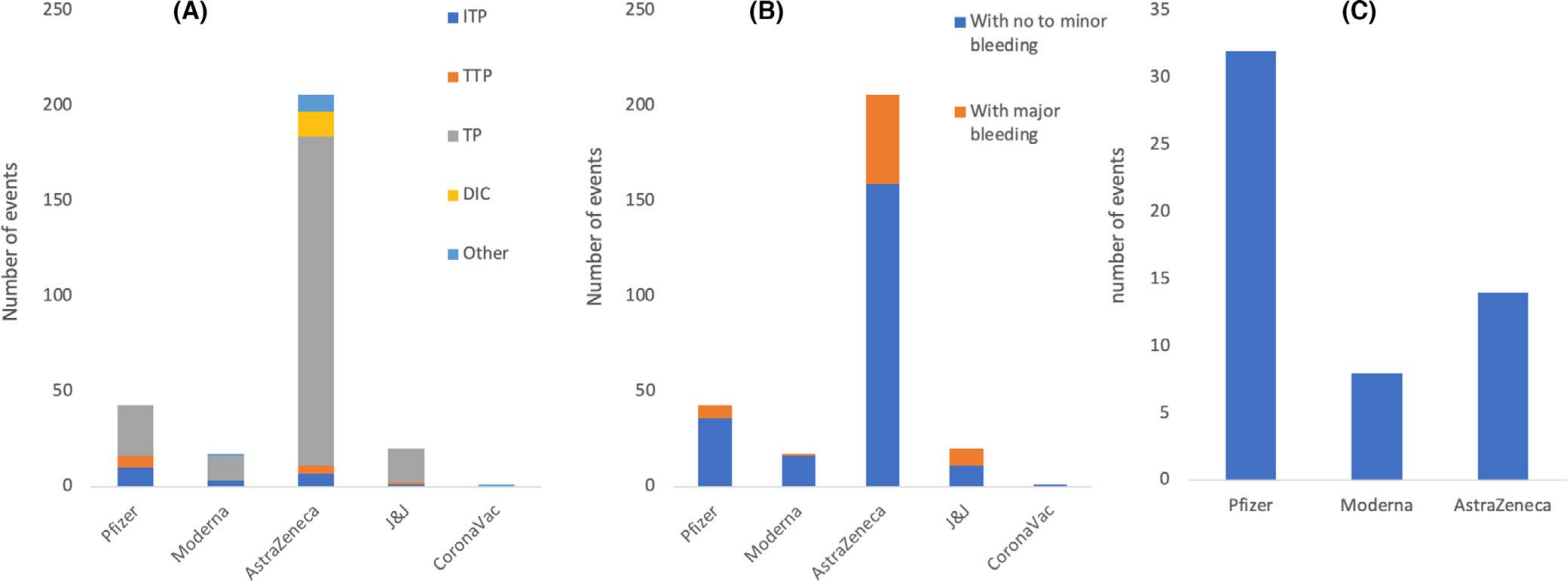
(A) Number of cases who had thrombocytopenia (TP) following COVID‐19 vaccination reported by 98 studies including Pottegård et al. and excluding Smadja et al. (reported separately). Immune thrombocytopenic purpura (ITP) was reported in 21 individuals post vaccination (10 Pfizer, 3 Moderna, 7 AstraZeneca and 1 J&J). A total of 11 thrombotic thrombocytopenic purpura (TTP) events (6 Pfizer, 4 AstraZeneca and 1 J&J) and 231 thrombocytopenia events (27 Pfizer, 13 Moderna 173 AstraZeneca and 18 J&J) were reported post vaccination. One case of familial thrombocytopenia flare was reported post Moderna vaccination and one case of haemophagocytic lymphohistiocytosis was reported after the CoronaVac vaccine. Thirteen cases of disseminated intravascular coagulation (DIC) and nine cases of vaccine‐induced thrombotic/pro‐thrombotic thrombocytopenia (VITT/VIPT) were reported following vaccination with AstraZeneca. Most of the cases of TP detected following AstraZeneca vaccination were associated with thrombosis. (B) Number of TP events classified as no to minor vs. major bleeding. A total of 223 TP events had no to minor bleeding (36 Pfizer, 16 Moderna, 159 AstraZeneca, 11 J&J and 1 CoronaVac) while a total of 64 TP events had major bleeding (7 Pfizer, 1 Moderna, 47 AstraZeneca and 9 J&J). Among Pfizer, 3 were ICH, 2 hematuria, 1 vaginal bleeding and 1 gastrointestinal (GI) bleeding. The case after Moderna was that of vaginal bleeding. Among AstraZeneca, 40 were ICH, 2 GI bleeding, 2 adrenal haemorrhage and 3 unspecified bleeding. As for J&J, 8 were ICH only and 1 had both ICH and mild retroperitoneal, intraperitoneal and pelvic haemorrhage. (C) Number of TP events following Pfizer (32), Moderna (8) and AstraZeneca (14) vaccination as reported by Smadja et al.

Figure 7A shows that ITP was reported in 21 individuals post vaccination (10 Pfizer, three Moderna, seven AstraZeneca and one J&J). A total of 11 TTP events (six Pfizer, four AstraZeneca and one J&J) and 231 thrombocytopenia events (27 Pfizer, 13 Moderna, 173 AstraZeneca and 18 J&J) were reported post vaccination. One case of familial TP flare was reported post Moderna vaccination and one case of haemophagocytic lymphohistiocytosis was reported after the CoronaVac vaccine. Thirteen cases of DIC and nine cases of vaccine‐induced thrombotic/pro‐thrombotic thrombocytopenia (VITT/VIPT) were reported following vaccination with AstraZeneca. A total of 223 TP events had no to minor bleeding (36 Pfizer, 16 Moderna, 159 AstraZeneca, 11 J&J and one CoronaVac), while a total of 64 TP events had major bleeding (seven Pfizer, one Moderna, 47 AstraZeneca and nine J&J). Among Pfizer, three were ICH, two hematuria, one vaginal bleeding and one GI bleed. The case after Moderna was that of vaginal bleeding. Among AstraZeneca, 40 were ICH, two GI bleeds, two adrenal haemorrhage and three unspecified bleeding. As for J&J, eight were ICH only and one had both ICH and mild retroperitoneal, intraperitoneal and pelvic haemorrhage. Smadja et al. reported 32 cases of thrombocytopenia associated to the venous and arterial thrombotic events after Pfizer vaccination while only eight and 14 cases were reported post Moderna and AstraZeneca vaccination (Figure 7B).

## 4 DISCUSSION

Our systematic review included 99 articles that reported the demographic and clinical data of at least 2567 cases (except those reported by Pottegård et al.) of CV and haematological complications following COVID‐19 vaccination. Association between such events and the five types of vaccines has not been confirmed, and some studies reported that some events may have coincided with the vaccine. In this review, we compiled all relevant complications which have been reported post COVID‐19 vaccination with a comprehensive discussion of the possible mechanisms without confirming their association with the vaccines. For example, MI and coronary artery thrombosis with an isolated thrombosis were each counted as cardiac events only as thrombosis can be caused by a plaque rupture rather than being induced by the vaccine. The review compares the CV and haematological events after receiving Pfizer, Moderna, AstraZeneca, J&J and CoronaVac vaccines. We have not specifically targeted those vaccines in our inclusion criteria. However, no relevant data were available for the other COVID‐19 vaccines.

It was observed that in some of the included studies, females represented more than 50% of the total affected cases. For example, Tobaiqy et al.^61^ reported that more female patients experienced thrombotic events at twice the rate that male patients did (n=19 women, n=9 men) which could be attributed to well‐established hormonal factors. It is well‐known that oral contraceptives increase the risk of thromboembolism in women of childbearing age. It has since been hypothesized that estrogen itself has prothrombotic effects although the exact mechanism has not been fully elucidated.^105^ Studies have shown that in the general population, when stratified by age, women generally have increased risk of thrombosis as compared to men of the same age, given their higher levels of estrogen.^106^ However, when the studies were compiled, there was no specific pattern for one gender being affected more than the other.

In all the included studies (except Smadja et al.), there were more cases of CV and haematological complications within the 35–54 age group in all five vaccines. The age was not specified for 47.4% of the 217 cases reported after AstraZeneca vaccination. However, most of the reported cases were 35–54 years old (49.1% of the reported cases). Smadja et al. reported the median ages for the cases with thrombosis following vaccination as 76, 72 and 67 years for Pfizer, Moderna and AstraZeneca, respectively. This contradicts what was widely argued about the AstraZeneca vaccine to cause thrombotic events in younger individuals.

In total, 1013 CV and haematological events were reported in at least 406 individuals who received Pfizer, Moderna and AstraZeneca vaccines as reported by 98 studies (Pottegård et al., was included in the number of events, but excluded from the number of cases as explained in the results section). The 1013 events were 14.9% CI, 45.4% thrombosis, 28.3% TP, 10.1% haemorrhage and 1.3% other CV and haematological events. Of these, 15.6, 4.4, 73.7, 6.0 and 0.2% were reported following Pfizer, Moderna, AstraZeneca, J&J and CoronaVac vaccines, respectively. This may indicate that the rate of occurrence of such adverse events is higher following AstraZeneca vaccination than the other COVID‐19 vaccines based on the data collected from 98 included studies. The same applies if we focus on the thrombotic events. A total of 460 thrombotic events were reported post COVID‐19 vaccination of which 9.8% were after Pfizer, 81.5% after AstraZeneca and 8.7% after J&J vaccination. This contradicts the results reported by Smadja et al., who obtained their data from VigiBase and reported 2245 arterial and/or venous thrombotic events with or without TP detected in 2161 patients following Pfizer, Moderna and AstraZeneca vaccination. Of these, 55.4%, 10.5% and 29.6% were reported following Pfizer, Moderna and AstraZeneca vaccines, respectively. According to Smadja et al., more thrombotic events were reported after Pfizer vaccine than with Moderna and AstraZeneca. However, the number of events following Moderna vaccine remains the lowest in the studies conducted by Smadja et al. and the remaining 98 included studies.

Many of the individuals who experienced CV and/or haematological complications post COVID‐19 vaccination had comorbidities/risk factors that may increase their chances to develop such complications or even could be the direct cause of such events. However, many other individuals developed such events post COVID‐19 vaccination without any underlying risk factors.

The details and proposed mechanisms of each type of event are described in the next sections.

### 4.1 Cardiac injury and hypertension

A total of 151 different events of CI and nine stage 2 (according to the new guidelines of the American Heart Association and the American College of Cardiology)^107^ HTN were reported by 98 studies while Smadja et al. reported 399 MI associated with thrombotic events post COVID‐19 vaccination.

Meylan et al.^45^ reported nine patients with stage 2 HTN (eight Pfizer and one Moderna) documented within minutes of vaccination. Eight of the nine patients had a history of HTN with most patients on antihypertensive therapy and reporting well‐controlled HTN.

Our results revealed that more myocarditis and myopericarditis events were reported after the mRNA vaccines, Pfizer and Moderna, while more MI and ischemic heart disease were mainly reported following the AstraZeneca vaccination.

### 4.2 How may vaccines induce hypertension or cardiac injury?

Our findings showed that the highest prevalence of myocarditis and myopericarditis was observed in individuals who received Pfizer and Moderna vaccines. While the overall rate of both side effects is low, it is important to look at the components of vaccines which may elicit such a response. mRNA vaccines contain polyethylene glycol (PEG). This lipid comes in numerous forms but serves the purpose of providing a lipophilic medium for active ingredients to enter cells and illicit an immune response.^108^ PEG is historically safe, with one meta‐analysis reporting on 37 case reports of anaphylaxis following exposure to PEG in different forms.^108^ It is possible that people who are allergic to PEG may develop an inflammatory response which may lead to myocarditis secondary to the allergic reaction. This may also explain the lower prevalence of myocarditis post AstraZeneca, J&J and Sinovac vaccines as they are devoid of PEG. One of the included studies described a case of Kounis Syndrome which is a type of coronary hypersensitivity reaction characterized by coronary‐like disease accompanied by systemic anaphylaxis.^109^ The patient in question had received the CoronaVac vaccine which contains potential allergen components, such as aluminum hydroxide.^110^

Another possible mechanism is the spike (S) protein of SARS‐CoV‐2 which binds with high affinity to angiotensin‐converting enzyme 2 (ACE2).^111^ ACE2 plays an important role in the renin–angiotensin–aldosterone system (RAAS), which is a hormonal system used to regulate blood pressure, electrolyte balance and systemic vascular resistance. Therefore, the interaction between the S protein and ACE2 may cause HTN due to the downregulation of ACE2. This may explain the cases of HTN following COVID‐19 vaccination as the vaccines work by introducing the S protein to the body to mount an immune response against it. Furthermore, ACE2 destroys angiotensin (AT) II into angiotensin (1–7) which modulates inflammation due to the inflammatory role of ATII.^112^ The interaction between COVID‐19 infection itself and the RAAS has been explored in depth in some of our previous studies, and the role of ACE2 in the inflammatory response remains one of the leading hypotheses explaining some of the phenomena observed with COVID‐19 infection and possibly now with vaccination as well.^113^, ^114^

COVID‐19–induced CI has been well‐established.^114^ Ammirati et al.,^6^ therefore, suggested that molecular mimicry between the SARS‐CoV‐2 viral proteins and cardiac molecules may partially explain the high incidence of CI observed during COVID‐19. Furthermore, an immuneresponse against the viral spike glycoprotein could pose a risk for immune‐mediated organ injury. Another explanation could be a nonspecific inflammatory response to some of the vaccine components.^115^ After conducting a case series of seven patients, Rosner et al.^10^ suggest that the clinical course of vaccine‐induced myocarditis is a favorable one with the resolution of symptoms in all patients. They believe that the risk benefit calculation is still strongly tipped in favor of vaccination.

The highest prevalence of MI was reported following the AstraZeneca vaccination. A few hypotheses have been created to establish the link between the AstraZeneca vaccine and MI. Greinacher et al.^71^ suggested that the vaccine may cause a post‐thrombotic state by inducing a thrombocytopenic purpura (TP) (resembling heparin‐induced TP). This may also explain the reported case of a healthy 54‐year‐old male who died after receiving the first dose of AstraZeneca vaccine. He was diagnosed with MI, PVT and TP.^59^ The same study reported two more cases of cardiac arrest and heart strain which were associated with PE and other thrombotic events and TP.^59^ It may also explain the 399 cases of MI that were associated with arterial thrombosis as reported by Smadja et al. Developing thrombotic events and TP post COVID‐19 vaccination will be discussed in the following sections. Boivin et al.^48^ suggested that the vaccine is not a causal but rather a contributing factor to the MI as the vaccination's side effects could be significant stressors that place increased demand on the heart, leading to demand ischemia. Merchant^116^ proposes the possibility of the transfection of platelets by mRNA or a viral vector‐based vaccine.

### 4.3 Haemorrhage

Radwi et al.,^46^ reported a case of acquired haemophilia A (AHA) in a 69‐year‐old male patient nine days after his second dose of the Pfizer vaccine. The patient suffered from diabetes, HTN, and adenocarcinoma of the prostate in remission. It is difficult to establish a link between AHA and the vaccine, but that it is plausible as the patient did not suffer from any conditions that are specifically linked with AHA, such as autoimmune disease. AHA has also been reported after the administration of H1N1 vaccine, the seasonal influenza vaccine and recently the Pfizer‐BioNTech SARS CoV‐2 mRNA vaccine.^46^ All three cases had low levels of factor VIII and the presence of FVIII inhibitors.^46^ Factor VIII is an essential component of the coagulation cascade that cleaves factor X in the presence of factor IX, and its deficiency causes haemophilia A.^117^ The mechanism underlying the development of AHA is unclear. It is proposed that certain T cell genetic polymorphisms may play a role in predisposing individuals to develop AHA.^118^ The mechanism of AHA might be similar to the molecular mimicry mechanism involved in ITP. Autoantibodies against factor VIII and activation of quiescent autoreactive T and B cells may play a role.^46^, ^119^, ^120^

A total of 102 haemorrhagic events were reported in the included studies, 26 of which were ICH. Many of the haemorrhagic events were associated with thrombosis. Thrombosis occurs when a blood clot is formed in blood vessels leading to decreased blood flow and certain implications including haemorrhage.^121^ Thrombosis occurs when there is a damage to the endothelial lining of the blood vessel, a hypercoagulable state or an arterial/venous blood statis. When damage to the blood vessel wall occurs, proinflammatory cytokines are activated, tissue factor availability is increased, adhesion molecules proliferate and platelets are activated.^121^ Thrombosis and haemorrhage are often viewed as two separate arms of the coagulation cascade. Too much activation of the cascade can cause an increased tendency to form thrombi and insufficient activation can cause bleeding tendencies and subsequent haemorrhage. There are a few pathological entities in which thrombosis and haemorrhage can occur together for specific pathophysiological mechanisms. The first of these is venous sinus thrombosis. It has been well‐described that patients with CVT often experience concurrent parenchymal haemorrhage of the brain. The proposed mechanism for this occurrence is that once the vessel gets blocked, there is a pressure build up that causes friable vessels to rupture leading to subsequent haemorrhage.^122^ The second instance in which such phenomena were observed together is in antineutrophil cytoplasmic antibodies (ANCAs)‐associated small vessel vasculitis (AAV). The vasculitis in these patients leads to vascular endothelial dysfunction which puts them at an increased risk of thrombosis.^123^ AAV is also commonly associated with haemorrhage in other organs, most commonly the lungs. This is also due to vascular dysfunction and subsequent rupture.^123^ This presents a unique dilemma for clinicians treating patients with AAV who present with thrombosis as well as concurrent pulmonary haemorrhage. Treatment with anticoagulation would seemingly worsen the pulmonary haemorrhage and lead to worse outcomes, whereas withholding anticoagulation can lead to a high thrombotic burden and worse outcomes.^123^

### 4.4 Thrombosis

Our results yielded 460 thrombotic events post COVID‐19 vaccination as reported by 98 studies in addition to 2161 events as reported by Smadja et al.

Our analysis revealed that CVST/CVT was the leading thrombotic complication comprising 34.6% of all thrombotic events. A retrospective cohort study investigated the occurrence of CVST in individuals receiving a COVID‐19 vaccine at the Mayo Clinic in the US. A total of 132916 COVID‐19 doses were administered (94819 Pfizer, 36352 Moderna and 1745 Janssen). Only three cases of CVST were reported within thirty days of the 1^st^ dose of the Pfizer vaccine for COVID‐19. One patient had a history of thrombosis while another had recent trauma. When analyzing data, it was discovered that the risk of CVST was similar when comparing 30 days post vaccination to 30 days prior to vaccination. Furthermore, the risk of CVST was also found to be similar when comparing 30 days post vaccination to 30 days of no vaccination or other vaccines. It was also concluded that the risk of CVST after receiving the first dose of the vaccine was similar to the baseline risk of CVST when looking at a large cohort in a multi‐state healthcare system. The old age of the patients who developed CVST following the Pfizer vaccine and the lack of concurrent TP may distinguish such cases from the VITT which were mainly reported in young females. Overall, the study did not report a significant association between COVID‐19 vaccination and the development of CVST.^24^

By considering that outside the veins of the lower extremities and pulmonary arteries are atypical, our results revealed that many of the thrombotic events occurred post COVID‐19 vaccination can be classified as atypical due to their location. In addition to CVT/CVST, which was the most common event to occur after vaccination among the venous thrombosis subcategory, the jugular vein was the most affected with Pfizer and J&J while the splanchnic vein was the most affected with AstraZeneca followed by the jugular vein. Other veins included in this subcategory were the iliac, hepatic, mesenteric, ophthalmic, inferior vena cava (IVC), azygous, epigastric, periuterin, femoral and brachial vein. Arterial thrombosis occurred less commonly than venous and included the following vessels: coronary, iliac, aorta, internal carotid, splenic, femoral, superior mesenteric, suprarenal, infrarenal, celiac and suprahepatic.

#### 4.4.1 How may vaccines induce thrombosis?

The mechanism of COVID‐19–induced coagulopathy is believed to overlap with that of DIC. The vaccine is believed to trigger a dysregulated immune response with excess release of inflammatory cytokines, increased amounts of damage‐associated molecular patterns and eventual activation of cell‐death mechanisms and vascular endothelial damage that led to the thrombophilic state^124^; therefore, standard anticoagulation therapy in patients receiving the COVID‐19 vaccine should be strongly recommended.^124^ More mechanisms are discussed in section 4.5 as more thrombotic events were associated with TP.

### 4.5 Thrombocytopenia

Our included studies reported 287 events of TP post COVID‐19 vaccination. The mechanism is explained in section 4.5 as many TP events were associated with thrombosis.

### 4.6 Thrombosis with thrombocytopenia

The majority of thrombotic events were associated with TP especially those reported post AstraZeneca vaccination.

#### 4.6.1 Thrombosis with thrombocytopenia post AstraZeneca vaccination

Scully et al.^59^ reported 23 patients with thrombosis (22 with CVT and one with haemorrhage) and TP 6 to 24 days after the first dose of the AstraZeneca vaccine. Anti‐PF4 antibodies were positive in 21 patients. Furthermore, Greinacher et al.^71^ reported 11 patients (36 median age) who had thrombotic complications post AstraZeneca vaccination, including CVT, splanchnic vein thrombosis, PE and other types of thrombi. Nine of these patients were found to have positive anti‐PF4 antibodies, and it was not assessed in the remaining two. Evidence of DIC was found in five of the patients as indicated by their elevated levels of D‐dimer as well as abnormalities in prothrombin time (PTT), INR and fibrinogen levels. Similarly, Mehta et al.^84^ described two young cases (32 and 25‐year‐old) of superior sagittal cerebral sinus venous thrombosis after the first dose of the AstraZeneca vaccine. One had no past medical history, while the second had a background of primary sclerosing cholangitis (PSC). Both patients had severe TP with low platelet count, and one had PF4 antibodies and factor V Leiden, and both rapidly deteriorated and succumbed to brain edema and herniation. Three more similar cases were reported by Wolf et al.^70^ (22, 36, 46‐year‐old women, previously healthy) who presented with intracranial venous sinus thrombosis (IVST) after receiving the 1^st^ dose of AstraZeneca. All three patients had low platelet count and positive anti‐PF4 antibodies suggesting HIT. The same applies to several other studies which reported various types of venous and arterial thrombosis with TP, including ischemic stroke, PE, DVT, pelvic vein thrombosis, peripheral artery thrombosis, CVT and carotid artery thrombosis post AstraZeneca vaccination.^61^, ^72^, ^88^

#### 4.6.2 How may vaccines induce thrombosis with thrombocytopenia?

Several mechanisms were proposed to explain the occurrence of VITT.

##### Antibodies PF4–polyanion complexes

Some of the patients with CVT were found to have high levels of antibodies to PF4–polyanion complexes.^72^ This resembles the underlying mechanism of heparin‐induced thrombocytopenia (HIT) when antibodies against the PF4‐heparin complex leads to the creation of a hypercoagulable state, resulting in arterial and venous thrombus formation and platelet depletion.^125^ Vaccine‐induced antibodies to a similar complex formed from a vaccine component and PF4 might have been responsible for the development of VITT. The vaccine component forming the complex against which antibodies are directed has still not been identified. However, some studies have investigated the potential cross‐reactivity of the anti–SARS‐CoV‐2 spike protein antibodies with PF4.^71^, ^72^, ^126^

##### Adenoviral vector and platelets

The adenoviral vector that forms the main component of the AZD1222 vaccine vector utilizes the coxsackie and adenovirus receptor (CAR).^127^ The CAR receptor facilitates viral entry into platelets.^128^ It could be possible that administration of the adenoviral vector vaccine could lead to occurrences of adenoviral particles in the blood that can bind to platelets and cause their aggregation.^126^, ^129^ This activation of circulating platelets leads to release of PF4 from the platelets.^125^ It can be possible that the adenoviral vector and platelet complex could itself lead to the induction of antibodies. As mentioned before, the complex of another unknown vaccine component and the released PF4 from the adenoviral vector–platelet interaction could also lead to autoantibody generation leading to VITT.^126^

##### Platelet expression of spike protein or adenoviral proteins

Another possible hypothesis proposed by Rzymski et al.^126^ includes the infection of platelets with the adenoviral vectors present in the vaccine. Plate precursors could get infected and transcribe the adenoviral DNA leading to spike protein or other adenoviral components being expressed on the platelet surface. This could theoretically lead to immune reactions and subsequent TP.^126^

#### 4.6.3 The astrazeneca controversy

After the rollout of the AstraZeneca vaccine across the European Union, reports started coming in of concerns of thrombotic events after vaccine administration. These included reports of CVST and sinus venous thrombosis (SVT) accompanied by TP. This led to several countries halting vaccine administration. On March, 2021, shortly after rollout, Austria suspended^126^ the use of a batch of of vaccines after two persons suffered blood clots after vaccination, one of whom died.^130^ This was followed by several other countries suspending the use of the vaccine including Norway, Germany, Canada, France, Italy and the Netherlands.^131^ By April, 2020, 169 cases of CVST and 53 cases of SVT were reported to EudraVigilance.^132^ The European medical agency reaffirmed the safety of the vaccine while listing blood clots with TP as a rare side effect.^132^ Following this, most countries resumed use of the vaccine while some restrained their use in younger individuals. The Netherlands, Denmark and Norway were an exception to this as they decided to permanently suspend the use of the vaccination.

The two mechanisms explained in sections 4.5.2.2 and 4.5.2.3 may explain the higher prevalence of thrombotic events with thrombocytopenia post AstraZeneca vaccination than Pfizer and Moderna as reported by the included studies (except Smadja et al.).

As explained previously, the S protein's interaction with ACE2 and the vaccine components each plays a role in what seems to be two separate responses in vulnerable patients. However, the current COVID‐19 vaccines provide recipients with both of these components, so it could be hypothesized that the combination of the two plays a role in inducing systemic inflammation leading to thrombosis and/or CI. However, it is important to note that the overall incidence of any severe adverse reaction to any COVID‐19 vaccine is still rare; so formulating and supporting a hypothesis is difficult.

In our primary data, we found the majority of thrombosis occurring in patients who took the AstraZeneca vaccine compared to the other vaccines in 98 included studies. The vector‐based vaccine works by delivering the DNA code for the spike protein, allowing our cells to transcribe it into mRNA, translate it into protein and mount an immune response against it.^133^ The mRNA vaccines deliver the mRNA code directly, requiring only translation from our cells to mount an immune response.^134^ In the vast majority of vaccines, the immune system creates numerous antibodies against the S protein of all COVID‐19 vaccines, protecting them from the real virus as well. It is when the immune system does not create high‐quality antibodies or when the S protein is altered that adverse events, such as thrombosis, may occur. One current hypothesis in the works states that the AstraZeneca viral vector allows for alternate splicing to occur, yielding different types of S proteins which are not recognized by antibodies against the default S protein, thus causing an inflammatory response.^135^ While still a very preliminary hypothesis, this may spearhead further research into the reasoning and reversal of such side effects.

In several patients with thrombosis and TP following the AstraZeneca vaccine, high levels of PF4 antibodies were observed without history of heparin exposure.^59^, ^70^, ^71^, ^72^, ^88^, ^136^ This VITT is still poorly understood. In normal HIT, an antigen complex of heparin and PF4 is created, activating the immune system to create an anti–heparin‐PF4‐complex antibody. This antibody will bind to the complex with the Fab region and bind to platelets with the Fc region, activating them and causing platelet aggregation.^136^ The reason for heparin binding to PF4 in the first place is due to the cationic charge of heparin and anionic charge of PF4. This understanding of HIT provides a basis toward understanding VITT following AstraZeneca vaccination.

### 4.7 How concerning are the cardiovascular and hematological events post‐COVID‐19 vaccination?

In order to answer this question, it is essential to evaluate the rate of incidence of such events following COVID‐19 vaccination. One limitation was that most of the included studies were either case series or case reports. It was, therefore, difficult to calculate the prevalence of the CV and haematological events among the vaccinated populations. However, it was possible to calculate the prevalence of the CI and thrombotic events from five population studies. For example, Pawlowski et al.^24^ reported that out of 132913 individuals who received the Pfizer vaccine, only three (0.002%) experienced thrombosis. Pottegård et al. reported that 0.0002% of 281264 individuals who received the AstraZeneca vaccine suffered from CI while 0.04% suffered from thrombosis. Similarly, Tobaiqy et al.^61^ reported 41 thrombotic events out of 54571 adverse events reported to the EU database. This means that thrombosis represented 0.075% of the reported adverse events following AstraZeneca vaccination. However, the number of individuals who received at least one dose of the AstraZeneca vaccine in the UK and EU was 17,000,000, suggesting a low prevalence of thrombosis following COVID‐19 vaccination (0.00024%). Sørvoll et al.^93^ reported that 1.62% of 492 people who received the AstraZeneca vaccine had TP. Finally, Smadja et al. reported a very low rate (0.0006% of 361734967) of thrombosis post Pfizer, Moderna and AstraZeneca vaccination. According to the National Health Service (NHS), UK^137^ the incidence of thrombosis after the second dose of AstraZeneca vaccination in the UK is 1.3 per million doses, and all cases were in patients aged 50 years or older.

In addition to the rarity of such events, it is important to understand that COVID‐19 itself may cause CI and thrombosis.^114^, ^138^ Furthermore, the rate of such severe events is much higher when associated with COVID‐19 than the rate of vaccine‐induced CI and thrombosis. For example, it was reported by Shi et al.^139^ that 19.7% of 416 hospitalized patients with COVID‐19 had CI while a meta‐analysis by Li et al.^140^ reported that at least 8.0% of the COVID‐19 patients experienced acute myocardial injury. Furthermore, while the prevalence of thrombosis was reported as 1.3 per million doses in the UK, the rate of the COVID‐19–associated thrombosis was reported as 22% (95% CI 0.08–0.40) in COVID‐19 patients which increased to 43% (95% CI 0.29–0.65) after admission to the intensive care unit.^141^ According to our study, we report a total of 1013 CV and haematological events as reported by 98 studies and 2161 cases as reported by Smadja et al following all types of COVID‐19 vaccines. This suggests that the rate of such events is approximately 0.000001% (3174/3.09 billion) as a total of 3.09 billion doses have been administered so far worldwide.^142^ Additionally, the CDC reports revealed that it was noted after compiling the data of the different adverse events reports that the occurrence of myocarditis and pericarditis was more apparent in the adolescent and younger populations. Much debate followed this observation, and many argued that the benefits of vaccinating this specific population en masse outweighed the assumed risk of developing cardiac complications. Many argued that this was especially true since people in this age population did not seem to be affected by COVID‐19 infection to the same extent as older people with extensive medical comorbidities. This prompted the Advisory Committee on Immunization Practices (ACIP) to explore this further, and it was extensively discussed in their public meetings. On June 23, 2021 after reviewing the available evidence, they determined that the benefits of vaccination greatly outweigh the risks even in adolescents and young adults.^143^

### 4.8 Recommendations

Castelli et al.^85^ reported that SARS‐CoV‐2 infection and vaccine has been associated with a high incidence of thromboembolic events. Physicians should have a high index of suspicion of thromboembolic events in patients who have been vaccinated with the COVID‐19 AstraZeneca vaccine. Greinacher et al.^71^ reported that in some patients, thromboses can develop at unusual sites such as the brain or abdomen, and clinical manifestations may become apparent 5 to 20 days after vaccination. If these manifestations are accompanied by TP, it may be an adverse effect of vaccination. Mehta et al.^84^ recommend a plain CT and a CT venogram in suspected patients, along with laboratory work‐up of coagulation factors and potential prothrombotic disorder work‐up, such as antiphospholipid, ADAMTS13 and HIT and factor V Leiden, which would put the patient at an increased baseline risk of thrombosis. In the context of the similarity of post COVID‐19 vaccination CSVT and HIT, it is recommended to perform a PF4 antibody ELISA, and Schultz, et al.^72^ recommended that physicians should have a low threshold for requesting ELISA. As far as management, the authors recommend avoiding heparin products in all forms, favoring instead the administration of IVIG. Meanwhile, many studies did not recommend altering vaccination programs in the context of reports of cases of incidental thromboses.^23^, ^25^ Furthermore, Pawlowski, et al.^24^ reported that none of the COVID‐19 vaccines has been associated with a statistically significant increased relative risk of CVST in the Mayo Clinic Health System. Mehta et al. reported that the benefits of vaccination outweigh the potential risks. Furthermore, the risk of thrombosis with COVID‐19 infection itself is high, especially if admitted to intensive care, highlighting further benefits of vaccination.

### 4.9 Limitations of the study

This study has some limitations, including the possible overlap between the reported cases among some studies, especially those that obtained their data from the same databases. In an attempt to overcome this problem, we separated the data extracted from the study by Smadja et al. as the study reported a high number of cases extracted from a database that was used by other studies without reporting the individual demographic and clinical data of each case. It was easier to detect and remove duplicates from the other studies that used the same databases whenever the individual demographic and clinical data were reported. One limitation was the small number of the cohort studies as the majority of the included studies were either case series or case reports. This did not allow enough data to calculate the rate of such events within a vaccinated population. Another limitation was the lack of evidence that any of the reported events were associated with or induced by the vaccines. For example, MI and coronary artery thrombosis with an isolated thrombosis were each counted as cardiac events only as thrombosis can be caused by a plaque rupture rather than being induced by the vaccine which cannot be confirmed without autopsy.

## 5 CONCLUSION

Like most medications and compounds that enter the human body, vaccines have long been associated with adverse events. These events are rare in occurrence and even more so in fatality. Vaccines at their core are made with the intention of stimulating the immune system. This may cause unintended activation or modulation of the immune system which can cause such things as cardiac injury or even development of antibodies against platelets; mechanisms which have been proposed to explain the reported adverse events. We are now experiencing the same thing with the COVID‐19 vaccines. A few reports of rare adverse reactions following vaccination, that are being attributed to the vaccines even though such an attribution, may not be true. This has caused a state of great hesitancy globally with many people reluctant to receive the vaccines, which may prove to be a hindrance to the global vaccine effort to slow the spread of COVID‐19. As such, it is important that the scientific community better characterizes these adverse events to the general population and provide appropriate recommendations to physicians.

The included studies (except Smadja et al.) revealed that the prevalence of CV and haematological events in general was higher following AstraZeneca vaccination. Furthermore, it was evident that more myocarditis and myopericarditis cases were reported following the mRNA vaccines, while more thrombotic events and haemorrhage were reported following the AstraZeneca vaccination. However, Smadja et al. revealed otherwise. One explanation to the discrepancy is the presence of bias in publishing or reporting the events following vaccination. Another possible explanation is the difference in the number of doses administered of each vaccine to date. As of April 30^th^, 2021, 127 million doses of the Pfizer/BioNtech vaccine, 104 million doses of the Moderna vaccine and 8 million doses of the Johnson&Johnson vaccine had been administered in the USA alone. At the same time across the world in Europe, 104 million doses of the Pfizer/BioNtech vaccine, 12 million doses of the Moderna vaccine and 28 million doses of the AstraZeneca vaccine had been administered. This discrepancy makes it difficult to really compare the different vaccines against each other. Moving forward, more studies are needed to assess the possible association between the COVID‐19 vaccines and the reported adverse CV and haematological events.

## CONFLICT OF INTEREST

The authors declare no conflict of interest.

## AUTHOR CONTRIBUTIONS

**Dana Al‐Ali:** Conceptualization (lead); Formal analysis (lead); Investigation (equal); Methodology (equal); Writing – review & editing (equal). **Abdallah Elshafeey:** Conceptualization (equal); Formal analysis (equal); Investigation (equal); Methodology (equal); Writing – original draft (lead). **Malik Mushannen:** Conceptualization (equal); Formal analysis (equal); Investigation (equal); Methodology (equal); Writing – original draft (lead); Writing – review & editing. **Hussam Kawas:** Conceptualization (equal); Formal analysis (equal); Investigation (equal); Methodology (lead); Writing – original draft (equal); Writing – review & editing (equal). **Ammena Shafiq:** Conceptualization (equal); Formal analysis (equal); Investigation (equal); Methodology (equal); Writing – original draft (equal); Writing – review & editing (equal). **Narjis Mhaimeed:** Formal analysis (equal); Investigation (equal); Methodology (equal); Writing – original draft (equal). **Nada Mhaimeed:** Conceptualization (equal); Formal analysis (equal); Investigation (equal); Methodology (equal); Writing – original draft (equal). **Omar Mahimeed:** Formal analysis (equal); Investigation (equal); Methodology (equal); Writing – original draft (equal). **Rached Zeghlache:** Investigation (equal); Methodology (equal); Writing – original draft (equal). **Mohammad Salameh:** Formal analysis (equal); Investigation (equal); Methodology (equal). **Pradipta Paul:** Formal analysis (equal); Investigation (equal); Methodology (equal). **Moayad Homssi:** Investigation (equal); Methodology (equal); Writing – original draft (equal). **Ibrahim Mohammed:** Formal analysis (equal); Investigation (equal); Methodology (equal). **Adeeb Narangoli:** Investigation (equal); Methodology (equal); Writing – original draft (equal). **Lina Yagan:** Formal analysis (equal); Investigation (equal); Methodology (equal). **Bushra Khanjar:** Formal analysis (equal); Methodology (equal). **Sa'ad Laws:** Investigation (equal); Methodology (equal). **Mohamed B. Elshazly:** Conceptualization (equal); Writing – review & editing (equal). **Dalia Zakaria:** Conceptualization (lead); Formal analysis (lead); Investigation (lead); Methodology (supporting); Project administration (lead); Writing – original draft (lead); Writing – review & editing (lead).

## Supporting information

AppendixS1

AppendixS2

Table S1

Table S2

Table S3

Table S4

Table S5

## Data Availability

The data that supports the findings of this study are available in the [Link], [Link] of this article.
