# Supplementary material for: Cardiovascular and haematological events post COVID‐19 vaccination: A systematic review
Source: J Cell Mol Med. 2021 Dec 29;26(3):636–53. doi: 10.1111/jcmm.17137 (PMC8817142; doi:10.1111/jcmm.17137)
Supplement: Supplementary file 1 — AppendixS1 [file JCMM-26-636-s001.docx]

The following databases were searched at the end of April 2021 (see appendix 1): PubMed, Medline (Ovid, 1946 – April 2021), Embase (Ovid, 1974 -2021), Scopus, Web of Science, Science Direct, MedRxiv, and Lens.org. All searches were limited by year to 2020 thru 2021 (or current date). All searches, where allowed, employed a combination of controlled vocabulary and keywords. A total of 48,292 articles were retrieved through initial searching. Results were imported into EndNote (version 19) and initial de-duplication was conducted using the Bramer methodology, which reduced the total number of articles to 19,736^.^ An additional 3,428 duplicates were removed when references were imported into Covidence, leaving 16,308 unique articles for initial screening. All searches were updated in July 2021 to include articles that were available after the initial searches. During extraction, the review team identified thrombocytopenia, thrombosis, myocarditis, and other similar terms as the focus of the final analysis of the review and as such, updated searches included these terms in addition to the base searches that were used initially. The updated searches resulted in 2,461 additional articles. The number of additional articles included in the update was reduced to 636 after deduplication was performed in Covidence.

**PubMed – Search run on April 26, 2021**

("severe acute respiratory syndrome coronavirus 2"[Supplementary Concept] OR severe-acute-respiratory-syndrome-coronavirus-2[Title/Abstract] OR 2019-ncov[Title/Abstract] OR 2019ncov[Title/Abstract] OR covid-19[Title/Abstract] OR covid19[Title/Abstract] OR covid2019[Title/Abstract] OR ncov2019[Title/Abstract] OR ncov-2019[Title/Abstract] OR hcov19[Title/Abstract] OR sars-cov-2[Title/Abstract] OR coronavirus[Title/Abstract] OR coronaviruses[Title/Abstract] OR corona-virus[Title/Abstract] OR corona-viruses[Title/Abstract] OR covid[Title/Abstract] OR hcov[Title/Abstract] or "Wuhan Coronavirus"[Title/Abstract] or "coronavirus"[MeSH Terms] or "COVID-19"[Mesh] or "SARS-CoV-2"[Mesh]) AND (Vaccin*[Title/Abstract] or "Vaccination"[Mesh] or "COVID-19 Vaccines"[Mesh])

Filters Used: Publication Date – Custom Range: 2020/1/1 – Current.

**Medline (Ovid, 1946 – April 2021) - Search run on April 27, 2021**

1 | (severe-acute-respiratory-syndrome-coronavirus-2 OR 2019-ncov OR 2019ncov OR covid-19 OR covid19 OR covid2019 OR ncov2019 OR ncov-2019 OR hcov19 OR sars-cov-2 OR coronavirus OR coronaviruses OR corona-virus OR corona-viruses OR covid OR hcov OR severe acute respiratory syndrome coronavirus 2).ti,ab.

2 | exp coronavirus/

3 | (Vaccin*).ti,ab.

4 | exp vaccination/ or exp COVID-19 Vaccines/

5 | 1 OR 2

6 | 3 OR 4

7 | 5 AND 6

8 | Limit 7 - 2020 to Current

**Embase (Ovid, 1974 – April 2021) - Search run on April 27, 2021**

1 | (severe-acute-respiratory-syndrome-coronavirus-2 OR 2019-ncov OR 2019ncov OR covid-19 OR covid19 OR covid2019 OR ncov2019 OR ncov-2019 OR hcov19 OR sars-cov-2 OR coronavirus OR coronaviruses OR corona-virus OR corona-viruses OR covid OR hcov OR severe acute respiratory syndrome coronavirus 2).ti,ab.

2 | exp coronavirus/

3 | (Vaccin*).ti,ab.

4 | exp vaccination/ or exp COVID-19 Vaccines/

5 | 1 OR 2

6 | 3 OR 4

7 | 5 AND 6

8 | Limit 7 - 2020 to Current

**Scopus - Search run on April 28, 2021**

1 | TITLE-ABS-KEY("severe-acute-respiratory-syndrome-coronavirus-2" OR "2019-ncov" OR "2019ncov" OR "covid-19" OR "covid19" OR "covid2019" OR "ncov2019" OR "ncov-2019" OR "hcov19" OR "sars-cov-2" OR "coronavirus" OR "coronaviruses" OR "corona-virus" OR "corona-viruses" OR "covid" OR "hcov" OR "severe acute respiratory syndrome coronavirus 2")

2 | INDEXTERMS(coronavirus OR SARS-CoV-2)

3 | TITLE-ABS-KEY(Vaccin*)

4 | INDEXTERMS ("COVID-19 Vaccines")

5 | 1 OR 2

6 | 3 OR 4

7 | 5 AND 6

Filters Used: Years limited to 2020 and 2021.

**Web of Science - Search run on April 26, 2021**

(TI=(severe-acute-respiratory-syndrome-coronavirus-2 OR 2019-ncov OR 2019ncov OR covid-19 OR covid19 OR covid2019 OR ncov2019 OR ncov-2019 OR hcov19 OR sars-cov-2 OR coronavirus OR coronaviruses OR corona-virus OR corona-viruses OR covid OR hcov OR severe acute respiratory syndrome coronavirus 2) OR AB=(severe-acute-respiratory-syndrome-coronavirus-2 OR 2019-ncov OR 2019ncov OR covid-19 OR covid19 OR covid2019 OR ncov2019 OR ncov-2019 OR hcov19 OR sars-cov-2 OR coronavirus OR coronaviruses OR corona-virus OR corona-viruses OR covid OR hcov OR severe acute respiratory syndrome coronavirus 2)) AND (TI=(vaccin*) OR AB=(vaccin*))

**Science Direct - Search run on April 26, 2021**

("severe-acute-respiratory-syndrome-coronavirus-2" OR covid19 OR coronavirus OR corona-viruses OR "severe acute respiratory syndrome coronavirus 2") AND (vaccine or vaccines or vaccination or vaccinations)

Filters Used: Years limited to 2020 and 2021

**MedRxiv - Search run on April 28, 2021**

Title and Abstracts searched for: "Covid 19 Vaccine" (match all words) and posted between "01 Jan, 2020 and 28 Apr, 2021"

**Lens.org - Search run on April 26, 2021**

("severe-acute-respiratory-syndrome-coronavirus-2" OR covid19 OR coronavirus OR corona-viruses OR "severe acute respiratory syndrome coronavirus 2") AND (vaccine or vaccines or vaccination or vaccinations)

Filters Used: Date – 2020 to Current, Flags - Cited By Scholarly Works

**Updated Searches**

**PubMed – Search run on July 5, 2021**

("severe acute respiratory syndrome coronavirus 2"[Supplementary Concept] OR severe-acute-respiratory-syndrome-coronavirus-2[Title/Abstract] OR 2019-ncov[Title/Abstract] OR 2019ncov[Title/Abstract] OR covid-19[Title/Abstract] OR covid19[Title/Abstract] OR covid2019[Title/Abstract] OR ncov2019[Title/Abstract] OR ncov-2019[Title/Abstract] OR hcov19[Title/Abstract] OR sars-cov-2[Title/Abstract] OR coronavirus[Title/Abstract] OR coronaviruses[Title/Abstract] OR corona-virus[Title/Abstract] OR corona-viruses[Title/Abstract] OR covid[Title/Abstract] OR hcov[Title/Abstract] or "Wuhan Coronavirus"[Title/Abstract] or "coronavirus"[MeSH Terms] or "COVID-19"[Mesh] or "SARS-CoV-2"[Mesh]) AND (Vaccin*[Title/Abstract] or "Vaccination"[Mesh] or "COVID-19 Vaccines"[Mesh]) AND (thrombocytopenia*[Title/Abstract] OR thromb*[Title/Abstract] OR "Blood Clot*"[Title/Abstract] OR hemophilia[Title/Abstract] OR myocarditis[Title/Abstract] OR "myocardial infract*"[Title/Abstract] OR "cardiac injur*"[Title/Abstract] OR hemorrha*[Title/Abstract] OR "Thrombosis"[Mesh] OR "Thrombocytopenia"[Mesh] OR "Myocarditis"[Mesh] OR "Myocardial Infarction"[Mesh] OR "Hemophilia A"[Mesh] OR "Hemophilia B"[Mesh] OR "Hemorrhage"[Mesh])

Filters Used: Publication Date – Custom Range: 2020/1/1 – Current.

**Medline (Ovid, 1946 – April 2021) - Search run on July 5, 2021**

1 | (severe-acute-respiratory-syndrome-coronavirus-2 OR 2019-ncov OR 2019ncov OR covid-19 OR covid19 OR covid2019 OR ncov2019 OR ncov-2019 OR hcov19 OR sars-cov-2 OR coronavirus OR coronaviruses OR corona-virus OR corona-viruses OR covid OR hcov OR severe acute respiratory syndrome coronavirus 2).ti,ab.

2 | exp coronavirus/

3 | (Vaccin*).ti,ab.

4 | exp vaccination/ or exp COVID-19 Vaccines/

5 | (thrombocytopenia* OR thromb* OR "Blood Clot*" OR hemophilia OR myocarditis OR "myocardial infract*" OR "cardiac injur*" OR hemorrha*).ti,ab.

6 | exp Thrombosis/ OR exp Thrombocytopenia/ OR exp Myocarditis/ OR exp Myocardial Infarction/ OR exp Hemophilia A/ OR exp Hemophilia B/ OR exp Hemorrhage/

7 | 1 OR 2

8 | 3 OR 4

9 | 5 OR 6

10 | 7 AND 8 AND 9

11 | Limit 10 to yr="2020 - Current"

**Embase (Ovid, 1974 – April 2021) - Search run on July 5, 2021**

1 | (severe-acute-respiratory-syndrome-coronavirus-2 OR 2019-ncov OR 2019ncov OR covid-19 OR covid19 OR covid2019 OR ncov2019 OR ncov-2019 OR hcov19 OR sars-cov-2 OR coronavirus OR coronaviruses OR corona-virus OR corona-viruses OR covid OR hcov OR severe acute respiratory syndrome coronavirus 2).ti,ab.

2 | exp coronavirus/

3 | (Vaccin*).ti,ab.

4 | exp vaccination/ or exp COVID-19 Vaccines/

5 | (thrombocytopenia* OR thromb* OR "Blood Clot*" OR hemophilia OR myocarditis OR "myocardial infract*" OR "cardiac injur*" OR hemorrha*).ti,ab.

6 | exp Thrombosis/ OR exp Thrombocytopenia/ OR exp Myocarditis/ OR exp Myocardial Infarction/ OR exp Hemophilia A/ OR exp Hemophilia B/ OR exp Hemorrhage/

7 | 1 OR 2

8 | 3 OR 4

9 | 5 OR 6

10 | 7 AND 8 AND 9

11 | Limit 10 to yr="2020 - Current"

**Scopus - Search run on July 5, 2021**

1 | TITLE-ABS-KEY("severe-acute-respiratory-syndrome-coronavirus-2" OR "2019-ncov" OR "2019ncov" OR "covid-19" OR "covid19" OR "covid2019" OR "ncov2019" OR "ncov-2019" OR "hcov19" OR "sars-cov-2" OR "coronavirus" OR "coronaviruses" OR "corona-virus" OR "corona-viruses" OR "covid" OR "hcov" OR "severe acute respiratory syndrome coronavirus 2")

2 | INDEXTERMS(coronavirus OR SARS-CoV-2)

3 | TITLE-ABS-KEY(Vaccin*)

4 | INDEXTERMS ("COVID-19 Vaccines")

5 | TITLE-ABS-KEY ("thrombocytopenia*" OR "thromb*" OR "Blood Clot*" OR "hemophilia" OR "myocarditis" OR "myocardial infract*" OR "cardiac injur*" OR "hemorrha*")

6 | INDEXTERMS (Thrombosis OR Thrombocytopenia OR Myocarditis OR Myocardial Infarction OR Hemophilia A OR Hemophilia B OR Hemorrhage)

7 | 1 OR 2

8 | 3 OR 4

9 | 5 OR 6

10 | 7 AND 8 AND 9

11 |( LIMIT-TO ( PUBYEAR , 2021 ) OR LIMIT-TO ( PUBYEAR , 2020 )

**Web of Science - Search run on July 5, 2021**

(TI=(severe-acute-respiratory-syndrome-coronavirus-2 OR 2019-ncov OR 2019ncov OR covid-19 OR covid19 OR covid2019 OR ncov2019 OR ncov-2019 OR hcov19 OR sars-cov-2 OR coronavirus OR coronaviruses OR corona-virus OR corona-viruses OR covid OR hcov OR severe acute respiratory syndrome coronavirus 2) OR AB=(severe-acute-respiratory-syndrome-coronavirus-2 OR 2019-ncov OR 2019ncov OR covid-19 OR covid19 OR covid2019 OR ncov2019 OR ncov-2019 OR hcov19 OR sars-cov-2 OR coronavirus OR coronaviruses OR corona-virus OR corona-viruses OR covid OR hcov OR severe acute respiratory syndrome coronavirus 2)) AND (TI=(vaccin*) OR AB=(vaccin*)) AND (TI=("thrombocytopenia*" OR "thromb*" OR "Blood Clot*" OR "hemophilia" OR "myocarditis" OR "myocardial infract*" OR "cardiac injur*" OR "hemorrha*") OR AB=("thrombocytopenia*" OR "thromb*" OR "Blood Clot*" OR "hemophilia" OR "myocarditis" OR "myocardial infract*" OR "cardiac injur*" OR "hemorrha*"))

Results limited by publication year to 2020 - 2021

**Science Direct - Search run on July 5, 2021**

(covid19 OR coronavirus OR "severe acute respiratory syndrome coronavirus 2") AND (vaccine OR vaccination) AND (thrombocytopenia OR thrombosis OR myocarditis OR "myocardial infraction")

Filters Used: Years limited to 2020 and 2021

**MedRxiv - Search run on July 5, 2021**

Title and Abstracts searched for: ""Covid 19 Vaccine" AND (thrombosis OR myocarditis OR "myocardial infraction")" and posted between "01 Jan, 2020 and 31 Dec, 2021"

**Lens.org - Search run on July 5, 2021**

("severe-acute-respiratory-syndrome-coronavirus-2" OR covid19 OR coronavirus OR corona-viruses OR "severe acute respiratory syndrome coronavirus 2") AND (vaccine or vaccines or vaccination or vaccinations) AND (thrombocytopenia OR thrombosis OR hemophilia OR myocarditis OR "myocardial infraction" OR "cardiac injury" OR hemorrhage)

Filters Used: Date – 2020 to Current, Flags - Cited By Scholarly Works
