# Supplementary material for: Cardiovascular and haematological events post COVID‐19 vaccination: A systematic review
Source: J Cell Mol Med. 2021 Dec 29;26(3):636–53. doi: 10.1111/jcmm.17137 (PMC8817142; doi:10.1111/jcmm.17137)
Supplement: Supplementary file 2 — AppendixS2 [file JCMM-26-636-s005.docx]

**Data Analysis**

CV and hematological events were classified into four major categories: cardiac injury (CI), thrombosis, thrombocytopenia (TP), and hemorrhage. Several cases had multiple events under different categories or within the same category. For this reason, 2 types of analyses were conducted: the number of cases who suffered from any type of CV and hematological events post-COVID-19 vaccination and the number of events under each category. For example, if one patient had three confirmed thrombotic events (pulmonary embolism (PE), deep vein thrombosis (DVT) and cerebral venous thrombosis (CVT)) and myocardial infraction (MI), in the first type of analysis, this will be counted as 1 case in each of cardiac and thrombosis, however, 3 events will be counted under thrombosis and 1 event under cardiac. Furthermore, many cases had multiple events under the same category. We classified each category into subcategories with another category for events that had to be reported separately but were too rare and did not occur across all vaccines to be classified under a separate category. The subcategories are as follows: CI; myocarditis, myopericarditis, MI, ischemic heart disease and other which included pericarditis, stress cardiomyopathy, acute coronary syndrome (ACS), heart strain, cardiac arrest and Kounis syndrome. Hemorrhage: intracranial cerebral hemorrhage (ICH), unspecified and other which included acquired hemophilia and uterine hemorrhage. Thrombosis: PE, cerebral venous (sinus) thrombosis (CVST/CVT), DVT, portal vein thrombosis (PVT), thrombotic thrombocytopenia (TTP), stroke and other which included blue toes, organ thrombosis/infarction, thrombophlebitis, thrombotic microangiopathy, transient ischemic attack (TIA) and unspecified. For each case, arterial or venous thrombotic (AT and VT respectively) events other than the previously specified were classified as 1 AT or VT even if multiple vessels were reported. Thrombocytopenia: idiopathic thrombocytopenia purpura (ITP), TTP, TP, disseminated intravascular coagulation (DIC) and other which included flare of familial thrombocytopenia and hemophagocytic lymphohistiocytosis. In cases where coronary artery thrombosis occurred as an isolated event, it was counted as a cardiac event only. However, if it occurred with other arterial thrombosis, it was counted as a thrombotic event under the arterial thrombosis subcategory. As for MI, we acknowledge that this event could occur due to thrombosis and/or plaque rupture. However, due to the inability to differentiate without an autopsy, MI cases were counted as cardiac only in our paper. Furthermore, bleeding/hemorrhage events occurring in the context of TP were not counted as separate events instead were considered as a sign/symptom of the TP. We further categorized these events into no to minor vs major bleeding where minor bleeding included epistaxis, blood blisters, hemorrhagic oral bullae and scleral hemorrhage while major bleeding included ICH, hematuria, vaginal bleeding, gastrointestinal (GI) bleeding and adrenal hemorrhage. Cases of suspected TP and hemorrhage were counted under both hemorrhage and TP with major bleeding.
