# Supplementary material for: Cardiovascular and haematological events post COVID‐19 vaccination: A systematic review
Source: J Cell Mol Med. 2021 Dec 29;26(3):636–53. doi: 10.1111/jcmm.17137 (PMC8817142; doi:10.1111/jcmm.17137)
Supplement: Supplementary file 3 — Table S1 [file JCMM-26-636-s004.docx]

**Supplementary Table 1:**  **Types of cardiovascular abnormalities in 104 adult and 18 children Pfizer vaccinated patients who developed cardiovascular disease in the included case reports/series.**

| **Type of Event** | **Event** | **N (Sex)** | **Age** | **Comorbidities** | **Which dose** | **Onset of symptoms** | **Signs and Symptoms** | **Diagnostic Method** | **Treatment** | **Outcome** | **References** | **Type of study and Country** |
| --- | --- | --- | --- | --- | --- | --- | --- | --- | --- | --- | --- | --- |
| **Cardiac only (46)** | Myocarditis | 1 (M) | 56 | COVID-19 infection but no CVD history | 2nd | 3 days | Chest pain | Troponin level cMRI | None | Recovered | Ammirati et al.^6^ | Case report, Italy |

|  | Myocarditis | 1 (M) | 23 | None | 2nd | 5 days | Severe chest pain, SOB, syncope/presyncope | ECG  Troponin level  cMRI | Corticosteroids  Colchicine | Recovered | Kim et al.^7^ | Case series, USA |
| --- | --- | --- | --- | --- | --- | --- | --- | --- | --- | --- | --- | --- |
|  | Myocarditis | 1 (M) | 24 | None | 2nd | 2 days | Severe chest pain, palpitations | ECG  Troponin level  cMRI | Colchicine NSAIDs | Recovered | Kim et al.^7^ | Case series, USA |
|  | Perimyocarditis | 1 (M) | 17.1 | None | 2nd | 3 days | Chest pain | ECG  Troponin level | Ibuprofen (5)  Aspirin (1)  None (1) | Recovered | Snapiri et al.^8^ | Case series, Israel |
|  | Perimyocarditis | 1 (M) | 16.2 | None | 2nd | 1 day | Chest pain | ECG  Troponin level |  | Recovered | Snapiri et al.^8^ | Case series, Israel |
|  | Perimyocarditis | 1 (M) | 16.8 | None | 2nd | 2 days | Chest pain, cough | ECG  Troponin level |  | Recovered | Snapiri et al.^8^ | Case series, Israel |
|  | Perimyocarditis | 1 (M) | 16.3 | None | 2nd | 3 days | Chest pain, nausea | ECG  Troponin level |  | Recovered | Snapiri et al.^8^ | Case series, Israel |
|  | Perimyocarditis | 1 (M) | 17.5 | None | 2nd | 1 day | Chest pain, headache | ECG  Troponin level |  | Recovered | Snapiri et al.^8^ | Case series, Israel |
|  | Perimyocarditis | 1 (M) | 16.6 | None | 1st | 2 days | Chest pain, dyspnea, diarrhea, fever | ECG  Troponin level |  | Recovered | Snapiri et al.^8^ | Case series, Israel |
|  | Perimyocarditis | 1 (M) | 17.6 | None | 2nd | 3 days | Chest pain, dyspnea | ECG  Troponin level |  | Recovered | Snapiri et al.^8^ | Case series, Israel |
|  | Myocarditis | 1 (M) | 24 | None | 2nd | 3 days | Chest pain radiating to the jaw | MRI | NSAIDs Colchicine | Recovered | Abu Mouche et al.^9^ | Case series, Israel |
|  | Myocarditis | 1 (M) | 20 | None | 2nd | 1 day | Chest discomfort | MRI | Ibuprofen Colchicine | Recovered | Abu Mouche et al.^9^ | Case series, Israel |
|  | Myocarditis | 1 (M) | 29 | None | 2nd | 2 days | Chest pain | MRI | NSAIDs Colchicine | Recovered | Abu Mouche et al.^9^ | Case series, Israel |
|  | Myocarditis | 1 (M) | 45 | HLD | 1st | 16 days | Chest pain | MRI | Ibuprofen Colchicine | Recovered | Abu Mouche et al.^9^ | Case series, Israel |
|  | Myocarditis | 1 (M) | 16 | None | 2nd | 1 day | Chest pain, muscle aches | MRI | Ibuprofen colchicine | Recovered | Abu Mouche et al.^9^ | Case series, Israel |
|  | Myocarditis | 1 (M) | 17 | None | 2nd | 3 days | Chest and abdominal pain | MRI | Ibuprofen Colchicine | Recovered | Abu Mouche et al.^9^ | Case series, Israel |
|  | Acute myocarditis-like illness | 1 (M) | 39 | NR | 2nd | 3 days | Sudden onset 7/10 chest pain associated with SOB worse when lying flat and with inspiration | Troponin level  ECG Echocardiogram  cMRI  Coronary angiography | Beta blocker  Angiotensin receptor blocker | Recovered | Rosner et al.^10^ | Case series, USA |
|  | Acute myocarditis-like illness | 1 (M) | 24 | NR | 1st | 7 days | Intermittent, positional chest pain, left arm numbness and tingling | Troponin level  ECG Echocardiogram  cMRI  Coronary angiography | Colchicine Ibuprofen Famotidine | Recovered | Rosner et al.^10^ | Case series, USA |
|  | Acute myocarditis-like illness | 1 (M) | 19 | NR | 2nd | 2 days | Mid-sternal sharp chest pain, waxing and waning and positional. Relieved with leaning forward. | Troponin level  ECG Echocardiogram  cMRI  Coronary angiography | Colchicine Ibuprofen Famotidine | Recovered | Rosner et al.^10^ | Case series, USA |
|  | Acute myocarditis-like illness | 1 (M) | 20 | Reported prior COVID-19 infection | 2nd | 3 days | Mid-sternal chest pain, with deep inspiration | Troponin level  ECG Echocardiogram  cMRI  Coronary angiography | Ibuprofen Famotidine | Recovered | Rosner et al.^10^ | Case series, USA |
|  | Acute myocarditis-like illness | 1 (M) | 23 | NR | 2nd | 3 days | Subjective fevers, diffuse myalgia and headache starting day of vaccination. Sudden onset of sharp chest pain the night prior to admission that persisted at 3/10 intensity, worse when lying flat | Troponin level  ECG Echocardiogram  cMRI  Coronary angiography | Beta Blocker  Colchicine | Recovered | Rosner et al.^10^ | Case series, USA |
|  | Myopericarditis | 1 (M) | 16 | Incidental benign coronary artery anomaly | 2nd | 12-24 hours | Myalgias, tactile fevers and sharp, stabbing mid-sternal chest pain radiating to left arm | ECG  Troponin level  CT Angiogram  cMRI | IVIG  Ibuprofen | Recovered | McLean et al.^11^ | Case report, USA |
|  | Acute myocarditis | 1 (M) | 17 | Episode of idiopathic acute myocarditis 4 months prior | 2nd | 24-48 hours | Fever and sudden onset of severe, burning left-sided chest pain radiating to the left shoulder and upper arm, body aches | Troponin  cMRI  ECG | NSAIDs  Supportive care | Recovered | Minocha et al.^12^ | Case report, USA |
|  | Myocarditis | 7 (M) | 20-51 | None | 1st (1)  2nd (6) | 12-96 hours | Acute onset  chest pain | ECG  Echocardiogram Troponin level* | Supportive care | Recovered or recovering | Montgomery et al.^13^ | Case series, USA |
|  | Myocarditis | 1 (M) | 16 | None | 2nd | 2 days | Fatigue, poor appetite,  chest pain, fever, pain in both arms | ECG  Troponin level  cMRI | IVIG  Methylprednisone  Prednisone | Recovered | Marshall et al.^14^ | Case series, USA |
|  | Myopericarditis | 1 (M) | 19 | None | 2nd | 3 days | Chest pain, myalgias, fatigue, fever | ECG  Troponin level  cMRI | Ketorolac  Colchicine  Aspirin | Recovered | Marshall et al.^14^ | Case series, USA |
|  | Myopericarditis | 1 (M) | 17 | None | 2nd | 2 days | Chest and left arm pain, parasthesias | ECG  Troponin level  Echocardiogram  cMRI | Ibuprofen | Recovered | Marshall et al.^14^ | Case series, USA |
|  | Myocarditis | 1 (M) | 18 | None | 2nd | 3 days | Chest pain, malaise, arthralgia, myalgia | ECG  Troponin level  Echocardiogram cMRI | IVIG Methylprednisolone Prednisone  Ibuprofen Aspirin | Recovered | Marshall et al.^14^ | Case series, USA |
|  | Myocarditis | 1 (M) | 17 | None | 2nd | 3 days | Chest pain, sore throat, dry cough, body aches, subjective fever | ECG  Troponin level  Echocardiogram cMRI | IVIG  Methylprednisolone Prednisone  Ibuprofen  Aspirin | Recovered | Marshall et al.^14^ | Case series, USA |
|  | Myocarditis | 1 (M) | 16 | None | 2nd | 3 days | Malaise, subjective fever, chest pain | ECG  Echocardiogram Troponin level  cMRI | IVIG  Prednisone  Morphine | Recovered | Marshall et al.^14^ | Case series, USA |
|  | Myopericarditis | 1 (M) | 14 | None | 2nd | 2 days | Fever, pleuritic chest pain | ECG  Echocardiogram Troponin level  cMRI | NSAIDs  Furosemide | Recovered | Marshall et al.^14^ | Case series, USA |
|  | Acute Myocarditis | 1 (M) | 37 | HTN | 2nd | 3 days | Severe chest pain, generalized body aches, fever, chills,  headache | cMRI  Troponin level  ECG | Dual antiplatelets therapeutic anticoagulation  Metoprolol  IV analgesics | Recovered | Habib et al.^15^ | Case report, Qatar |
|  | Myocarditis | 1 (M) | 15 | Mild intermittent asthma (in one patient but did not specify which one). | 1st | 3 days | Acute onset, mid-sternal, non-radiating chest pain associated with chest tightness | Troponin level  CK-MB  NT-proBNP  ECG  Echocardiogram | One patient received IVIG, while the other patient improved without treatment (but did not specify which one) | Recovered with mild ST elevation at discharge | Park et al.^16^ | Case series, USA |
|  | Myocarditis | 1 (M) | 16 |  | 2nd | 2 days | Acute onset, mid-sternal, non-radiating chest pain associated with chest tightness | Troponin T  CK-MB  NT-proBNP  ECG  Echocardiogram  cMRI |  | Recovered with mild ST elevation at discharge | Park et al.^16^ | Case series, USA |
|  | Myocarditis | 1 (M) | 19 | None | 2nd | 4 days | Acute substernal chest pressure, SOB | ECG  Troponin level  CRP  Transthoracic echocardiogram  cMRI  Coronary angiograph  ESR | Lisinopril Metoprolol succinate | Recovered | Vidula et al.^17^ | Case series, USA |
|  | Stress Cardiomyopathy | 1 (F) | 60 | Stent placed in LAD 3 years ago | 2nd | 4 days | Exertional chest pain | ECG  Troponin level  Transthoracic echocardiogram  Coronary angiography | Metoprolol succinate Lisinopril | Recovered | Vidula et al.^17^ | Case series, USA |
|  | Pericarditis | 1 (F) | 21 | ITP | 1st | 21 days | Chest pain worsened with inspiration and while supine | ECG  Troponin level  CRP  Transthoracic echocardiogram | Colchicine | Recovered | Vidula et al.^17^ | Case series, USA |
|  | Pericarditis | 1 (F) | 61 | HTN | 2nd | 28 days | Low-grade fevers, night sweats, chest discomfort, palpitations | ECG  Troponin level  CRP Transthoracic echocardiogram  ESR | Colchicine | Recovered | Vidula et al.^17^ | Case series, USA |
|  | Myocarditis | 1 (M) | 30 | None | 2nd | 3 days | Dyspnea, constrictive retrosternal pain, nausea, profuse sweating | cMRI | Bisoprolol Acetylsalicylic acid Prednisolone | Recovered | D’angelo et al.^18^ | Case report, Italy |
|  | STEMI  Triple coronary artery thrombosis (first diagonal branch and distal part of the LAD, and the right coronary artery) | 1 (M) | 86 | Prostate cancer  AFib | 1st | 30 minutes | Collapse | ECG  Coronary angiogram | Percutaneous coronary intervention with manual aspiration thrombectomy  Balloon angioplasty Eptifibatide | Died | Tajstra et al.^19^ | Case report, Poland |
| **Cardiac with thrombosis (1)** | MI  PE | 1 (M) | Elderly | Apoplexy  MI  HTN  DM2  Dementia | NR | 2 days | None | Forensic autopsy | None | Died | Edler et al.^20^ | Case series, Germany |
| **Cardiac and thrombocytopenia with no to minor bleeding (1)** | Myocarditis Thrombocytopenia | 1 (F) | 44 | NR | NR | Same day | Nausea, vomiting, chest pain | NR | NR | NR | Lee et al.^21^ | Case series, USA |
| **Cardiac, thrombosis and thrombocytopenia with no to minor bleeding (1)** | MI  PE  Thrombocytopenia | 1 (F) | 82 | NR | NR | NR | Dyspnea, neutropenia | NR | NR | Died | Welsh et al.^22^ | Case series, USA |
| **Thrombosis only (12)** | DVT | 1 (F) | 66 | Post traumatic left leg neuropathy | 2nd | 2 days | Right calf pain | Doppler US | Apixaban | Recovered | Carli et al.^23^ | Case report, Italy |
|  | CVST | 3 (2F/1M) | 79-84 | 7 (1 localized cancer, 2 heart disease, 1 mild to moderate liver disease, 1 pulmonary disease, 1 thrombosis and 1 recent trauma) | NR | Within 30 days | NR | Manual review by clinicians of patients identified as positive for CVT by artificial intelligence | NR | NR | Pawlowski et al.^24^ | Retrospective cohort study, USA |
|  | PE | 1 (F) | Elderly | CHD  Cardiac insufficiency HTN  Dementia Hyperthyroidism Emphysema  Hiatal hernia Pseudomembranous colitis  Leg dermatitis | 1st | 3 days | Fever | Forensic autopsy | Antipyretic as needed | Died | Edler et al.^20^ | Case series, Germany |
|  | Micro thrombosis in small arterioles | 1 (M) | Elderly | CKD  Anemia  AFib  PE  HTN  PAD  Stroke  Epilepsy gait disorder with polyneuropathy  RA  Prostate carcinoma with prostatectomy Pancreatitis | 1st | 7 days | Respiratory insufficiency (pneumonia) | Forensic autopsy | Hospital admission | Died | Edler et al.^20^ | Case series, Germany |
|  | External jugular vein thrombophlebitis | 1 (F) | 55 | NR | 2nd | 2 days | Left supraclavicular lump, malaise, ipsilateral axillary lymphadenopathy | US  CT | NR | NR | Martinez et al.^25^ | Case report, Spain |
|  | Blue toes | 1 (F) | 41 | Bipolar disorder on valproate for >10 years | 1st | 4 days | Sudden toe pain with walking impairment and itching at night | Diagnosis of exclusion | Apixaban Low-dose aspirin | Recovered | Davido et al.^26^ | Case report, France |
|  | CVST (SSS extending to torcula herophili, left transverse sinus, and sigmoid sinus to proximal internal jugular vein) | 1 (M) | 49 | Childhood asthma  CAD  Allergy to seafood and nonsteroidal medication | 1st and 2nd | 16 days | Headache, giddiness | CT cerebral venography | Clexane Clopidogrel | Improved | Zakaria et al.^27^ | Case report, Malaysia |
|  | PE | 1 (M) | 27 | None | 2nd | 6 days | Chest pain | CT angiogram | Enoxaparin followed by apixaban | Discharged on apixaban | Abu Esba et al.^28^ | Case series, Saudi Arabia |
|  | DVT  PE | 1 (F) | 59 | DM  Osteoarthritis  COVID-19 pneumonia in 09/2020  OCP use for 20 years | 1st | 7 days | Sudden-onset left leg pain | Duplex US  CT angiogram | Enoxaparin followed by rivaroxaban | Discharged | Al-Miqbali et al.^29^ | Case report, Oman |
|  | CVT/CVST (superior sagittal, right  transverse and sigmoid sinuses and cortical vein)  Jugular vein thrombosis** | 1 (F) | 67 | Multiple cerebral cavernous malformations  HTN  DM  DLP  Viral myocarditis  Depression  Possible RCC found on work up | 2nd | 3 days | Right lower limb clonic movements, followed by motor deficit, loss of consciousnes, headache, tongue bite | Brain MRI | Levitaracetam Enoxaparin Dabigatran | Recovered | Dias et al.^30^ | Case series, Portugal |
| **Thrombosis and thrombocytopenia with no to minor bleeding (5)** | PE Thrombocytopenia | 1 (NR) | NR | NR | NR | NR | NR | NR | NR | Died | Lee et al.^21^ | Case series, USA |
|  | TTP relapse | 1 (F) | 48 | Relapsing TTP | 2nd | 6 days | Ecchymoses on both arms and forearms | ADAMTS13 level/activity  Platelet count Clinical picture | Plasma exchange Corticosteroids | Recovered | Sissa et al.^31^ | Case report, Italy |
|  | Acquired TTP | 1 (M) | 28 | Morbid obesity | 2nd | 28 days | Dysarthria, chest pain | ADAMTS13 level/activity  Antibody levels | Plasma exchange  High dose steroids  Caplacizumab  Rituximab | Recovered | Mayaan et al.^32^ | Case series, Israel |
|  | Acquired TTP | 1 (M) | 30 | Episode of acquired TTP in 2013 | 2nd | 8 days | Purpura on limbs | ADAMTS13 level/activity  Antibody levels | Plasma exchange  High dose steroids  Caplacizumab  Rituximab | Recovered | Mayaan et al.^32^ | Case series, Israel |
|  | Immune-mediated TTP | 1 (F) | 38 | None | 1st | 14 days | Spontaneous bruising, petechiae, blurred vision in the left eye with increased bruising after 2nd dose | Platelet count ADAMTS13 level/activity Antibody titers | Plasmapheresis with fresh frozen plasma  Methylprednisolone  Low-dose acetylsalicylic acid  Caplacizumab | Recovered | de Bruijn et al.^33^ | Case report, Belgium |
| **Thrombosis and thrombocytopenia with major bleeding (3)** | CVST (right straight and sigmoid sinuses)  Jugular vein thrombosis  Suspected PE  Thrombocytopenia | 1 (F) | 55 | Obesity | 2nd | 1 day | Headache, fever, brain hemorrhage | NR | Enoxaparin | Died | Ciccone et al.^34^ | Case series, Italy |
|  | Acquired TTP | 1 (F) | 40 | None | 2nd | 8 days | Somnolence,low-grade fever, petechiae, ecchymosis, macroscopic hematuria | ADAMTS13 level/activity  Antibody levels | Plasma exchange  High dose steroids  Caplacizumab | Recovered | Mayaan et al.^32^ | Case series, Israel |
|  | Acquired TTP | 1 (F) | 31 | Recurrent acquired TTP in remission | 1st | 13 days | Vaginal bleeding, purpura | ADAMTS13 level/activity  Antibody levels | Plasma exchange  High dose steroids  Caplacizumab  Rituximab | Continuing treatment with Caplacizumab | Mayaan et al.^32^ | Case series, Israel |
| **Thrombosis with suspected thrombocytopenia @ (9)** | CVT % | 8 (NR) | 20-89 | NR | NR | NR | NR | NR | NR | NR | Schulz et al.^35^ | Case series, Germany |
|  | Ischemic Stroke **%%** | 1 (NR) | 31-82 | NR | 1st | NR | NR | NR | NR | Died (2) | Schulz et al.^35^ | Case series, Germany |
| **Thrombosis with hemorrhage/bleeding (4)** | CVST (transverse and sigmoid sinuses)  Jugular vein thrombosis  ICH (temporo-parietal intraparenchymal hemorrhage) | 1 (M) | 54 | HLD | 2nd | 1-2 days | Severe headache, vomiting followed by acute left hemiparesis | CT brain  CT venogram | Decompressive craniectomy Unfractionated heparin  followed by LMWH | Rehabilitation | Fan et al.^36^ | Case series, Singapore |
|  | CVST (transverse and sigmoid sinuses)  Jugular vein thrombosis  PE #  Arterial thrombosis (left internal iliac artery) #  Venous thrombosis  (right common iliac vein thrombi) #  ICH (temporo-occipital hemorrhage and SAH) | 1 (F) | 62 | HTN | 2nd | 9 days | Headache, vomiting | CT venogram | Decompressive craniectomy Unfractionated heparin  followed by LMWH with bridging to warfarin | NR | Fan et al.^36^ | Case series, Singapore |
|  | CVT/CVST (transverse and sigmoid sinuses and bilateral cortical veins)  Jugular vein thrombosis  ICH (right occipital hematoma, bilateral SAH and perirolandic gyri infarcts) | 1 (F) | 60 | DM  HTN  HLD | 2nd | 8 days | Right ataxic hemiparesis | CT brain and CT venogram | LMWH followed by bridging to warfarin | Recovered | Fan et al.^36^ | Case series, Singapore |
|  | CVT/CVST (superior sagittal,  right lateral, transverse and sigmoid sinuses and cortical venous infarct)  Jugular vein thrombosis  ICH (right frontal SAH) | 1 (F) | 47 | Iron deficiency anemia due to adenomyosis  On OCP | 1st | 6 days | Headache, nausea, photophobia, left motor defect, papilledema, left visual extinction, right gaze deviation, left hemiparesis | Brain MRI | Acetazolamide  Enoxaparin  Warfarin | Resolved except for a slight gait instability | Dias et al.^30^ | Case series, Portugal |
| **Thrombocytopenia with no to minor bleeding (20)** | Thrombocytopenia | 1 (F) | 36 | None | 1st | 5 days | Weakness, menorrhagia, blood blisters, petechiae, epistaxis | Platelet count | NR | NR | Lee et al.^21^ Welsh et al.^22^ | Case series, USA |
|  | Thrombocytopenia | 1 (M) | 41 | ITP in 2012  DM1  Hypothyroidism  GERD | 1st | 2 days | Epistaxis, petechiae | Platelet count | Platelet transfusion  IVIG  Prednisone  Rituximab | Discharged | Lee et al.^21^ Welsh et al.^22^ | Case series, USA |
|  | Thrombocytopenia | 1 (M) | 73 | HTN  DM  HLD  Hypothyroidism | 1st | 1 day | Petechiae,  bruising | Platelet count | Corticosteroids  IVIG  Platelet transfusion | Improved | Lee et al.^21^ Welsh et al.^22^ | Case series, USA |
|  | Thrombocytopenia | 1 (M) | 53 | Fatty Liver  HLD  HTN | 1st | 15 days | None (found on routine physical) | Platelet count | Corticosteroids  Platelet transfusion  IVIG | Improved | Lee et al.^21^ Welsh et al.^22^ | Case series, USA |
|  | Thrombocytopenia | 1 (F) | 41 | Neuropathy | 1st | 3 days | Chest pain,  rash on breast, waist and thigh | Platelet count | Corticosteroids  IVIG | Improved | Lee et al.^21^ | Case series, USA |
|  | Thrombocytopenia | 1 (M) | 53 | Crohn’s disease  HTN  GERD  Pre-DM  Nephrolithiasis | 1st | 7 days | Petechiae, hemorrhagic oral bullae | Platelet count | Corticosteroids  IVIG | Improved | Lee et al.^21^ Welsh et al.^22^ | Case series, USA |
|  | Thrombocytopenia | 1 (M) | 59 | None | NR | NR | NR | NR | NR | NR | Lee et al.^21^ Welsh et al.^22^ | Case series, USA |
|  | ITP | 1 (M) | 74 | HTN | 1st | 1 day | Hemorrhagic blisters on oral and nasal mucosa, purpuric rash  on lower extremities | Platelet count | Dexamethasone  Platelet transfusion | NR | Krajewski et al.^37^ | Case report, Poland |
|  | ITP | 1 (M) | 53 | Morbid obesity  DM  HTN  Suspected otitis 1 week prior to admission (Took 2 tablets of levofloxacin) | 1st | 14 days | Epistaxis, wet  purpura on palate,  petechial and purpuric rash on the trunk and limbs | Platelet count | Dexamethasone   IVIG | Recovered | Ganzel et al.^38^ | Case report, Israel |
|  | ITP | 1 (F) | 41 | Multiple  allergies (quinolones, cephalosporins, strawberries  and iodinated contrast)  Hypothyroidism  HTN  pre-DM | 1st | 12 hours | Fever,  tachycardia, nausea, malaise, headache, loose stools on multiple  occasions later developed headache, gum bleeding, petechiae | Platelet count | IV fluids  Analgesic Antipyretic  Methylprednisolone  Dexamethasone  IVIG | Recovered | Fueyo-Rodriguez et al.^39^ | Case report, Mexico |
|  | ITP | 1 (F) | 54 | Congenital epidermal dysplasia  HTN  Overactive bladder  Mild cognitive impairment  CKD  Anxiety | 1st | 7 days | Progressive diffuse non-pruritic painless petechial rash in the lower extremities, chest and abdomen, increased mucosal bleeding, worsening ecchymosis | Platelet count | Dexamethasone  Platelet transfusion  IVIG | Recovered | Idogun et al.^40^ | Case report, USA |
|  | ITP | 1 (M) | 84 | Essential tremor Localized bladder cancer  Mild CKD  Paroxysmal AFib | 1st | 5 days | Widespread petechiae, especially on lower extremities, gum bleeding, limbs bruising. | Platelet count  Anti-platelet antibodies  (GPIIb-IIIa) | Platelet transfusion Prednisone  IVIG | Improved | Pasin et al.^41^ | Case report, Italy |
|  | Thrombocytopenia | 1 (NR) | NR | NR | NR | NR | Reported decrease in platelets after vaccination | NR | NR | NR | Welsh et al.^22^ | Case series, USA |
|  | Thrombocytopenia | 1 (F) | 39 | Depression  PCOS | 2nd | 2 days | Diffuse petechiae, increased menstrual bleeding | Platelet count | Platelet transfusion Solumedrol Prednisone IVIG | In-hospital | Welsh et al.^22^ | Case series, USA |
|  | Thrombocytopenia | 1 (F) | 78 | AFib  Essential tremor Thyroid nodule | 1st | 6 days | Petechiae | Platelet count | IVIG Dexamethasone  Platelet transfusion | Discharged | Welsh et al.^22^ | Case series, USA |
|  | Thrombocytopenia | 1 (F) | 55 | HTN  DM  Arthritis | 1st | 4 days | Petechiae, gum sores | Platelet count | Dexamethasone  IVIG  Platelet transfusion | In-hospital | Welsh et al.^22^ | Case series, USA |
|  | ITP flare | 1 (F) | 28 | ITP | 1st and 2nd | 3 days | Petechiae, ecchymoses | Platelet count | Prednisone  IVIG | Improved | Kuter et al.^42^ | Case series, USA |
|  | ITP flare | 1 (F) | 71 | ITP | 1st and 2nd | 4 days | Petechiaa, ecchymoses, fatigue | Platelet count | Prednisone | Improved | Kuter et al.^42^ | Case series, USA |
|  | ITP flare | 1 (F) | 34 | ITP  Prior DVT (on warfarin) | 1st and 2nd | 2 days | Petechiae, ecchymoses, oral blood blisters | Platelet count | Prednisone  IVIG | Improved | Kuter et al.^42^ | Case series, USA |
|  | ITP flare | 1 (F) | 85 | ITP  AFib (on warfarin) | 1st and 2nd | 2 days | Petechiae | Platelet count | NR | Improved | Kuter et al.^42^ | Case series, USA |
| **Thrombocytopenia with major bleeding (4)** | Thrombocytopenia | 1 (M) | 56 | None | 1st | 3 days | Generalized purpura and petechiae, gingival bleeding, cerebral and  scleral hemorrhage | Platelet count | Corticosteroids  IVIGs  Platelet and RBC transfusion  Rituximab  Eltrombopag  Cyclosporin  Emergency craniectomy and splenectomy | Died | Lee et al.^21^ Welsh et al.^22^ | Case series, USA |
|  | ITP | 1 (M) | 22 | Upper respiratory tract infection 2 months prior | 1st | 3 days | Petechiae, epistaxis, gingival/gum bleeding, scleral hemorrhage, hematuria | Platelet count | Corticosteroids  IVIG  Platelet transfusion | Recovered | Lee et al.^21^ Welsh et al.^22^ Tarawneh et al.^43^ | Case series, USA |
|  | Thrombocytopenia | 1 (F) | 39 | HBV | 1st | 12 days | Bruising, bleed in the brain that led to a stroke | Platelet count | Platelet and RBC transfusion  Unspecified brain surgery | Recovered/Discharged to rehabilitation | Lee et al.^21^ Welsh et al.^22^ | Case series, USA |
|  | Thrombocytopenia | 1 (M) | 80 | Recent transcatheter aortic valve  replacement due to aortic stenosis Hypothyroidism  Diverticulosis  HTN  HLD  DM | NR | 6 days | Bloody diarrhea/GI bleed | Hemoglobin level  Platelet count | Platelet and RBC transfusion | Improved | Lee et al.^21^ Welsh et al.^22^ | Case series, USA |
| **Hemorrhage/bleeding only (4)** | ICH | 1 (F) | 61 | None | 1st | 3 days | None (found dead) | Spinal tap showed bloody CSF | None (found dead) | Died | Shimazawa et al.^44^ | Case series, Japan |
|  | ICH (left cerebellopontine hematoma and secondary SAH) | 1 (F) | 26 | None | 1st | 4 days | None (found dead) | Postmortem imaging | None (found dead) | Died | Shimazawa et al.^44^ | Case series, Japan |
|  | ICH (large  hematoma with ventricular rupture) | 1 (F) | 72 | Hepatitis C DLP | 1st | 3 days | Dysarthria, headache, nausea | Imaging | NR | Died | Shimazawa et al.^44^ | Case series, Japan |
|  | ICH | 1 (F) | 69 | None | 1st | 9 days | None (found dead) | Autopsy | None (found dead) | Died | Shimazawa et al.^44^ | Case series, Japan |
| **HTN (8)** | Stage 2 HTN | 1 (M) | 54 | HTN | 1st | Minutes | Malaise | Oscillometric manometer | Transfer to ER Amlodipine | Recovered | Meylan et al.^45^ | Case series, Switzerland |
|  | Stage 2 HTN | 1 (F) | 87 | HTN | 1st | Minutes | Malaise | Oscillometric manometer | Transfer to ER | Recovered | Meylan et al.^45^ | Case series, Switzerland |
|  | Stage 2 HTN | 1 (F) | 65 | HTN  Coiled vascular aneurysm with requirement to maintain SBP under 140 mm Hg | 1st | Minutes | Headache | Oscillometric manometer | Transfer to ER  Nifedipine | Recovered | Meylan et al.^45^ | Case series, Switzerland |
|  | Stage 2 HTN | 1 (F) | 70 | HTN | 1st | Minutes | Chest pain | Oscillometric manometer | Transfer to ER | Recovered | Meylan et al.^45^ | Case series, Switzerland |
|  | Stage 2 HTN | 1 (M) | 88 | White coat HTN | 1st | Minutes | Diaphoresis | Oscillometric manometer | None | Recovered | Meylan et al.^45^ | Case series, Switzerland |
|  | Stage 2 HTN | 1 (F) | 77 | HTN | 1st | Minutes | Anxiety | Oscillometric manometer | Refused monitoring and workup | Recovered | Meylan et al.^45^ | Case series, Switzerland |
|  | Stage 2 HTN | 1 (F) | 73 | HTN | 1st | Minutes | Tingling in mouth | Oscillometric manometer | Self-medication with nifedipine | Recovered | Meylan et al.^45^ | Case series, Switzerland |
|  | Stage 2 HTN | 1 (F) | 55 | NR (No HTN) | 1st | Minutes | Malaise  Headache | Oscillometric manometer | Transfer to ER  Amlodipine Monitoring Transition to outpatient care | Recovered | Meylan et al.^45^ | Case series, Switzerland |
| **Acquired Hemophilia**  **(1)** | AHA | 1 (M) | 69 | DM  HTN  Prostate adenocarcinoma in remission | 1st and 2nd | Symptom 1: 9 days after 1^st^ dose  Symptom 2: after 2^nd^ dose (onset NR) | Symptom 1: mild bruising on left wrist  Symptom 2: several new bruises on arms and legs with one expanding, associated with swelling following minor trauma | Coagulation profile MRI | High dose prednisone | Recovered | Radwi et al.^46^ | Case report, Saudi Arabia |
| **Microangiopathy (3)** | Purpuric lesions on both eyelids | 1 (F) | 44 | NR | 2nd | 21-25 days | Asymptomatic lesions circumscribed on the upper eyelid | Visual inspection | None | Recovered | Mazzatenta et al.^47^ | Case series, Italy |
|  | Purpuric lesions on both eyelids | 1 (M) | 63 | NR | 2nd | 21 days | Asymptomatic lesions circumscribed on the upper eyelid | Visual inspection | None | Recovered | Mazzatenta et al.^47^ | Case series, Italy |
|  | Ecchymotic lesions on upper eyelids | 1 (F) | 67 | NR | 1st | 10 days | Moderately itchy lesions | Visual inspection | None | Recovered | Mazzatenta et al.^47^ | Case series, Italy |

*Among all cases reported in this study (including some who took Moderna), 8 patients had cMRI with T2 weighting, 16 patients underwent coronary artery imaging while all had an ECG, echocardiography and elevated troponins.

** Patient also had findings of focal polymorphic delta slowing, located in the right frontal-temporal region, and epileptiform discharges on EEG.

@The case series does not separate the age-range, symptoms, first or second dose, and the patients’ recovery, between the different vaccines of Pfizer and Astrazeneca; instead they were all grouped together. More information may be found in Supplementary Table 1 of the article. The study also did not specify which cases had thrombocytopenia.

% The rate of thrombocytopenia associated with CVT in this study was 60.5%

%% The rate of thrombocytopenia associated with Ischemic Stroke in this study was 33.3%

# Event occurred 3 weeks after admission

**ADAMTS13**: A Disintegrin and Metalloproteinase with a Thrombospondin type 1 motif, member 13; **AFib**: Atrial Fibrillation; **AHA**: Acquired Hemophilia; **CAD**: Coronary Artery Disease; **CHD**: Coronary Heart Disease; **CK-MB**: Creatine Kinase-Myocardial Band; **CKD**: Chronic Kidney Disease; **cMRI**: Cardiac Magnetic Resonance Imaging; **CRP**: C-Reactive Protein; **CT**: Computed Tomography; **CVD**: Cardiovascular Disease ; **CVST**: Cerebral Venous Sinus Thrombosis; **CVT**: Cerebral Venous Thrombosis; **DLP**: Dyslipidemia; **DM**: Diabetes Mellitus; **DM1**: Type 1 Diabetes Mellitus; **DM2**: Type 2 Diabetes Mellitus; **DVT**: Deep Vein Thrombosis; **ECG**: Electrocardiogram; **EEG**: Electroencephalogram; **ER**: Emergency Room; **ESR**: Erythrocyte Sedimentation Rate; **F**: Female; **GERD**: Gastroesophageal Reflux Disease; **GI**: Gastrointestinal; **HBV**: Hepatitis B Virus; **HLD**: Hyperlipidemia; **HTN**: Hypertension; **ICH**: Intracerebral Hemorrhage; **ITP**: Immune Thrombocytopenic Purpura; **IV**: Intravenous; **IVIG**: Intravenous Immune Globulin; **LAD**: Left Anterior Descending Artery; **LMWH**: Low Molecular Weight Heparin; **M**: Male; **MI**: Myocardial Infarction; **MRI**: Magnetic Resonance Imaging; **NR**: Not Reported; **NSAIDs**: Non-Steroidal Anti-Inflammatory Drugs; **NT-proBNP**: N-terminal pro B-type Natriuretic Peptide; **OCP**: Oral Contraceptive Pills; **PAD**: Peripheral Artery Disease; **PCOS**: Polycystic Ovarian Syndrome; **PE**: Pulmonary Embolism; **RA**: Rheumatoid Arthritis; **RBC**: Red Blood Cell; **RCC**: Renal Cell Carcinoma; **SAH**: Subarachnoid Hemorrhage; **SBP**: Systolic Blood Pressure; **SOB**: Shortness of Breath; **SSS**: Superior Sagittal Sinus; **STEMI**: ST Elevation Myocardial Infarction; **TTP**: Thrombotic Thrombocytopenic Purpura; **US**: Ultrasound
