# Supplementary material for: Cardiovascular and haematological events post COVID‐19 vaccination: A systematic review
Source: J Cell Mol Med. 2021 Dec 29;26(3):636–53. doi: 10.1111/jcmm.17137 (PMC8817142; doi:10.1111/jcmm.17137)
Supplement: Supplementary file 4 — Table S2 [file JCMM-26-636-s007.docx]

**Supplementary Table 2: Types of cardiovascular abnormalities in 44 adult Moderna vaccinated patients who developed cardiovascular disease in the included case reports/series.**

| **Type of Event** | **Event** | **N (Sex)** | **Age** | **Comorbidities** | **Which dose** | **Onset of symptoms** | **Signs and Symptoms** | **Diagnostic Method** | **Treatment** | **Outcome** | **References** | **Study and Country** |
| --- | --- | --- | --- | --- | --- | --- | --- | --- | --- | --- | --- | --- |
| **Cardiac only (25)** | MI | 1 (F) | 96 | Uncontrolled HTN | 1st | 1 hour | Chest discomfort | ECG  Echocardiogram  Troponin level | Heparin | Recovered | Boivin et al.^48^ | Case report, USA |
|  | Acute Myocarditis | 1 (M) | 36 | None | 2nd | 3 days | Severe chest pain, SOB | ECG  Troponin level  cMRI | Colchicine NSAIDs | Recovered | Kim et al.^7^ | Case series, USA |
|  | Acute Myocarditis | 1 (F) | 70 | HTN  Hypercholesterolemia | 2nd | 1 day | Severe chest pain, SOB, diaphoresis | ECG  Troponin level  cMRI | None | Recovered | Kim et al.^7^ | Case series, USA |
|  | Acute myocarditis-like illness | 1 (M) | 39 | NR | 2nd | 4 days | Fever, chills, SOB, chest heaviness/pain symptoms | Troponin level  ECG Echocardiogram  CXR  cMRI  Coronary angiography | IV steroids | Recovered | Rosner et al.^10^ | Case series, USA |
|  | Myocarditis | 1 (M) | 52 | HTN  Hypercholesterolemia  OSA  Possible hepatic steatosis (minor elevations in LFTs) | 2nd | 3 days | Substernal chest pain, high fevers, shaking chills, myalgias, headache | ECG  Echocardiogram  Troponin level  Coronary angiography  cMRI with contrast | Low dose  lisinopril  Carvedilol | Recovered | Muthukumaret al.^49^ | Case report, USA |
|  | Myocarditis | 16 (M) | 20-51 | None | 1st (2)  2nd (14) | 12-96 hours | Acute onset  chest pain | ECG  Echocardiogram Troponin level* | Supportive Care | Recovered or recovering | Montgomery et al.^13^ | Case series, USA |
|  | Myocarditis | 1 (M) | 25 | None | 2nd | 1 day | Subjective fever, chills,  substernal chest pain | ECG  Troponin level  cMRI  Echocardiogram  Coronary angiography | NR | Recovered | Mansour et al.^50^ | Case series, USA |
|  | Myocarditis | 1 (F) | 21 | None | 2nd | 1-2 days | Lightheadedness during usual exercise routine, chest pain radiating to left jaw | ECG  Troponin level  cMRI  Pulmonary angiogram  Echocardiogram | Metoprolol | Recovered | Mansour et al.^50^ | Case series, USA |
|  | Myocarditis | 1 (M) | 18 | None | 2nd | 1 day | Fevers, myalgias, acute substernal chest pain | ECG  Troponin level  CRP  Transthoracic echocardiogram  cMRI Coronary CT angiograph  ESR | Metoprolol succinate Colchicine Ibuprofen | Recovered | Vidula et al.^17^ | Case series, USA |
|  | Myocarditis | 1 (M) | 24 | None | 2nd | 1-4 days | Subjective fever, chills, body ache, substernal chest pain | Troponin level  cMRI | Beta blocker | Discharged | Albert et al.^51^ | Case report, USA |
| **Thrombocytopenia with no to minor bleeding**  **(16)** | Severe refractory ITP | 1 (M) | 74 | HTN  Gout  HLD  NICM | 1st | 1 day | Acute epistaxis, diffuse cutaneous purpura | Platelet count  Peripheral blood smear | Dexamethasone  IVIG  Platelet  transfusions  Rituximab  TPO-RA Eltrombopag  Plasma exchange  Methylprednisolone  Romiplostim | Recovered | Helms et al.^52^ | Case report, USA |
|  | Thrombocytopenia | 1 (F) | 25 | Anxiety  Anti-thyroglobulin Abs | 1st | 10 days | Oral mucosal bleeding, diffuse petechiae, ecchymoses | Platelet count | Corticosteroids  IVIG | Recovered | Lee et al.^21^ Welsh et al.^22^ | Case series, USA |
|  | Thrombocytopenia | 1 (F) | 43 | GERD | 1st | 8 days | Diffuse petechiae, bruising | Platelet count | IVIG  Prednisone | Discharged | Lee et al.^21^ Welsh et al.^22^ | Case series, USA |
|  | Thrombocytopenia | 1 (F) | 26 | None | 1st | 2 days | Bruising | Platelet count | Corticosteroids  IVIG  Platelet transfusion | Improved | Lee et al.^21^ Welsh et al.^22^ | Case series, USA |
|  | Thrombocytopenia | 1 (F) | 72 | Gout  DM | 1st | 1 day | Petechiae, bruises,  blood blisters | Platelet count | Corticosteroids  IVIG  Rituximab  Amicar  Vincristine  Romiplostim | Improved | Lee et al.^21^ | Case series, USA |
|  | Thrombocytopenia | 1 (F) | 50 | HTN | 1st | 23 days | Petechiae | Platelet count | Corticosteroids  IVIG  Platelet transfusion | Improved | Lee et al.^21^ Welsh et al.^22^ | Case series, USA |
|  | Flare of familial thrombocytopenia | 1 (F) | 36 | Inherited thrombocytopenia/ ITP | 1st | 7-16 days | 7 days post 1^st^ dose: Headaches 14 days post 1^st^ dose: Diffuse petechiae of the extremities/trunk, oral ecchymosis | Platelet count  Peripheral smear | Corticosteroids  IVIG | Recovered | Lee et al.^21^  Welsh et al.^22^  Toom et al.^53^ | Case report/series, USA |
|  | Thrombocytopenia | 1 (NR) | NR | NR | 1st | 1 day | Headache, “blister in head officially shingle” | Platelet count | Corticosteroids | Improved | Lee et al.^21^ Welsh et al.^22^ | Case series, USA |
|  | Thrombocytopenia | 1 (F) | 38 | ITP in 2019 Antiplatelet Abs | 2nd | 2 days | Headache, muscleach, petechiae | Platelet count | Corticosteroids  IVIG | Improved | Lee et al.^21^ Welsh et al.^22^ | Case series, USA |
|  | Thrombocytopenia | 1 (M) | 63 | HTN  DM  HLD | 1st | 11 days | NR | Platelet count | “Typical ITP therapies” | No response to therapy, in-hospital | Lee et al.^21^ Welsh et al.^22^ | Case series, USA |
|  | Thrombocytopenia | 1 (M) | 36 | Epilepsy | 1st | 15 days | NR | Platelet count | NR | NR | Lee et al.^21^ Welsh et al.^22^ | Case series, USA |
|  | Thrombocytopenia | 1 (M) | 37 | NR | NR | 4 days | NR | NR | Medications  Platelet transfusion | Recovered | Lee et al.^21^ Welsh et al.^22^ | Case series, USA |
|  | ITP | 1 (F) | 72 | Gout  DM2  Contact dermatitis | 1st | 1 day | Rash, spontaneous oral bleeding, headache | Clinical diagnosis | Dexamethasone  IVIG  Aminocaproic acid  Rituximab | NR | Julian et al.^54^ | Case report, USA |
|  | ITP flare | 1 (M) | 34 | Highly refractory ITP  Asplenic | 1st | 3 days | Petechiae, ecchymoses | Platelet count | NR | Improved | Kuter et al.^42^ | Case series, USA |
|  | Thrombocytopenia | 1 (F) | 72 | NR | 1st | 1 day | Blood blisters in mouth, diffuse bruising | Platelet count | Unspecified steroids | NR | Welsh et al.^22^ | Case series, USA |
|  | Thrombocytopenia | 1 (F) | 49 | Migraines  Psoriasis | 1st | 1 day | Petechiae, SOB | Platelet count | NR | NR | Welsh et al.^22^ | Case series, USA |
| **Thrombocytopenia with major bleeding (1)** | Thrombocytopenia | 1 (F) | 48 | HTN  Obesity | 1st | 13 days | Heavy vaginal bleeding | Platelet count | Corticosteroids  IVIG  Platelet transfusion | Recovered | Lee et al.^21^ Welsh et al.^22^ | Case series, USA |
| **HTN (1)** | Stage 2 HTN | 1 (F) | 87 | HTN | 1st | Minutes | Swelling at injection site | Oscillometric manometer | Transfer to ER  Candesartan Isosorbide dinitrate  Monitoring Transition to outpatient care | Recovered | Meylan et al.^45^ | Case series, Switzerland |
| **HTN and hemorrhage (1)** | Hypertensive crisis  ICH (left basal ganglia) | 1 (F) | 71 | NR | 1st | 3 days | Patient collapsed without any previous symptom then developed right hemiplegia, aphasia, agnosia | CT brain | IV clonidine hydrochloride  IV furosemide | Died | Athyros et al.^55^ | Case report, Greece |

*Among all cases reported in this study (including some who took Pfizer), 8 patients had cMRI with T2 weighting, 16 patients underwent coronary artery imaging while all had an ECG, echocardiography and elevated troponins.

**Abs**: Antibodies; **cMRI**: Cardiac Magnetic Resonance Imaging; **CRP**: C-Reactive Protein; **CT**: Computed Tomography; **CXR**: Chest X-Ray; **DM**: Diabetes Mellitus; **DM2**: Type 2 Diabetes Mellitus; **ECG**: Electrocardiogram; **ER**: Emergency Room; **ESR**: Erythrocyte Sedimentation Rate; **F**: Female; **GERD**: Gastroesophageal Reflux Disease; **HLD**: Hyperlipidemia; **HTN**: Hypertension; **ICH**: Intracerebral Hemorrhage; **ITP**: Immune Thrombocytopenic Purpura; **IV**: Intravenous; **IVIG**: Intravenous Immune Globulin; **LFTs**: Liver Function Tests; **M**: Male; **MI**: Myocardial Infarction; **NICM**: Nonischemic Cardiomyopathy; **NR**: Not Reported; **NSAIDs**: Non-Steroidal Anti-Inflammatory Drugs; **OSA**: Obstructive Sleep Apnea; **SOB**: Shortness of Breath; **TPO-RA**: Thrombopoietin Receptor Agonist
