# Supplementary material for: Cardiovascular and haematological events post COVID‐19 vaccination: A systematic review
Source: J Cell Mol Med. 2021 Dec 29;26(3):636–53. doi: 10.1111/jcmm.17137 (PMC8817142; doi:10.1111/jcmm.17137)
Supplement: Supplementary file 5 — Table S3 [file JCMM-26-636-s002.docx]

**Supplementary Table 3: Types of cardiovascular abnormalities in >217 adult AstraZeneca vaccinated patients who developed cardiovascular disease in the included case reports/series.**

| **Type of Event** | **Event** | **N (Sex)** | **Age** | **Comorbidities** | **Which dose** | **Onset of symptoms** | **Signs and Symptoms** | **Diagnostic Method** | **Treatment** | **Outcome** | **References** | **Study and Country** |
| --- | --- | --- | --- | --- | --- | --- | --- | --- | --- | --- | --- | --- |
| **Cardiac only**  **(>4)** | STEMI | 1 (M) | 63 | NR | 1st | 2 days | Chest pain, dizziness, excessive sweating | ECG  CK-MB Troponin level  Echocardiogram | Streptokinase  Anti-platelets  Anti-anginal drugs | Recovered | Chatterjee et al.^56^ | Case report, India |
|  | ACS (three vessel disease: LAD, distal left circumflex and right coronary artery) | 1 (M) | 46 | HTN  DM | 2nd | 12 days | Dyspnea, acute persistent cough | Troponin level  ECG  Coronary angiogram | Anti-platelets  Statins  Retelpase Percutaneous coronary intervention with stent placement | Recovered | Srinivasan et al.^57^ | Case series, India |
|  | ACS (double vessel disease: LAD and left circumflex) | 1 (M) | 48 | HTN  DM  CAD | 2nd | 6 days | Severe chest pain, cough, sweating | ECG  Coronary angiogram | Antiplatelet  Statin  Stent placement | Recovered | Srinivasan et al.^57^ | Case series, India |
|  | ACS (three vessel disease: posterior-lateral branch, LAD and small 1st branch of obtuse marginal) | 1 (F) | 75 | NR | 1st | 1 day | Central chest blocking sensation, profuse sweating | Troponin level  ECG  Coronary angiogram | Anti-platelet  Statin  Anti-anginal drugs | Recovered | Srinivasan et al.^57^ | Case series, India |
|  | MI | 20 ¶ | 32-55 | NR | 1st | NR | NR | NR | NR | NR | Pottegård et al.^58^ | Cohort study, Denmark and Norway |
|  | Ischemic heart disease without MI | 46 ¶ | 32-55 | NR | 1st | NR | NR | NR | NR | NR | Pottegård et al.^58^ | Cohort study, Denmark and Norway |
| **Cardiac, thrombosis and thrombocytopenia with no to minor bleeding (3)** | Heart strain  Extensive bilateral PE Thrombocytopenia | 1 (M) | 38 | NR | 1st | 14 days | NR | NR | NR | Died | Scully et al.^59^ | Case series, UK |
|  | Cardiac arrest Saddle PE  DVT Thrombocytopenia | 1 (F) | 70 | NR | 1st | 17 days | NR | NR | NR | Alive | Scully et al.^59^ | Case series, UK |
|  | MI  PVT  Thrombocytopenia | 1 (M) | 54 | NR | 1st | 10 days | NR | NR | NR | Died | Scully et al.^59^ | Case series, UK |
| **Cardiac, thrombosis, and thrombocytopenia with major bleeding (1)** | MI  CVT/CVST (SSS thrombosis, vein of Galen)  Multi-district thromboses including the aortic arch  DIC | 1 (F) | 54 | Meniere’s disease | NR | 12 days | Multiple subacute intra-axial hemorrhages in atypical locations, including the right frontal and temporal lobes with ipsilateral hemorrhagic subarachnoid suffusion | CT brain  ECG  Brain MRI | Plain old balloon angioplasty of the right coronary artery | Died | D’Agostino et al.^60^ | Case report, Italy |
| **Thrombosis**  **(>26)** | DVT | 12  (8F/4M) | 18-64 (8)  65-85 (3)  85+ (1) | NR | NR | NR | Malaise, pyrexia, purpura, cellulitis, lymphadenitis | NR | NR | Recovered (7)  Not resolved (2)  NR (3) | Tobaiqy et al.^61^ | Retrospective descriptive study, Multinational |
|  | PE  DVT | 4 (F) | 18-64 (2)  85+ (2) | NR | NR | NR | Lethargy, chest pain, fatigue,  pain in extremities | NR | NR | Recovered (1)  Died (2)  NR (1) | Tobaiqy et al.^61^ | Retrospective descriptive study, Multinational |
|  | Pelvic vein thrombosis | 1 (F) | 18-64 | NR | NR | NR | NR | NR | NR | Not resolved | Tobaiqy et al.^61^ | Retrospective descriptive study, Multinational |
|  | Thrombophlebitis | 2 (M) | 18-64 | NR | NR | NR | NR | NR | NR | Recovered (1)  Not resolved (1) | Tobaiqy et al.^61^ | Retrospective descriptive study, Multinational |
|  | Unspecified thrombosis | 4 (2F/2M) | 18-64 (3)  65-85 (1) | NR | NR | NR | Chest discomfort and pain | NR | NR | Recovered (2)  NR (1)  Not resolved (1) | Tobaiqy et al.^61^ | Retrospective descriptive study, Multinational |
|  | CVST (superior sagittal and transverse sinuses thrombosis with venous collaterals) | 1 (M) | 51 | None | 1st | 6 days | persistent holocranial headache, vomiting, double vision, loss of parallelism | MR venography | LMWH | Recovered | Dutta et al.^62^ | Case report, India |
|  | CVST (inferior sagittal, anterior part of the superior sagittal, left straight and sigmoid sinuses thrombosis) | 1 (M) | 64 | None | 1st | 0 days | NR | NR | Enoxaparin  IV mannitol Throbmectomty Craniectomy | No neurological deficit on day 20 | Ciccone et al.^34^ | Case series, Italy |
|  | CVT | 7 ¶ | 32-55 | NR | 1st | NR | NR | NR | NR | NR | Pottegård et al.^58^ | Cohort study, Denmark and Norway |
|  | PE | 21 ¶ | 32-55 | NR | 1st | NR | NR | NR | NR | NR | Pottegård et al.^58^ | Cohort study, Denmark and Norway |
|  | DVT (lower limb thrombosis) | 22 ¶ | 32-55 | NR | 1st | NR | NR | NR | NR | NR | Pottegård et al.^58^ | Cohort study, Denmark and Norway |
|  | Splanchnic thrombosis | <5 ¶ | 32-55 | NR | 1st | NR | NR | NR | NR | NR | Pottegård et al.^58^ | Cohort study, Denmark and Norway |
|  | Other thrombosis (Includes embolism and thrombosis in non-specified veins, other specified veins, and in the caval vein) | 12 ¶ | 32-55 | NR | 1st | NR | NR | NR | NR | NR | Pottegård et al.^58^ | Cohort study, Denmark and Norway |
|  | Cerebral Infarction | 16 ¶ | 32-55 | NR | 1st | NR | NR | NR | NR | NR | Pottegård et al.^58^ | Cohort study, Denmark and Norway |
|  | PE | 1 (M) | 77 | NR | 1st | 8 days | NR | NR | NR | Alive | Scully et al.^59^ | Case series, UK |
| **Thrombosis and thrombocytopenia with no to minor bleeding (>77)** | Atypical thrombosis: CVT, splanchnic and other thrombosis DIC | 1 (M) | 41 | NR | NR | 17 days | NR | NR | NR | Died | Gras-Champel et al.^63^ | Case series. France |
|  | Atypical thrombosis: CVT, splanchnic and other thrombosis DIC | 1 (M) | 63 | NR | NR | 11 days | NR | NR | NR | Died | Gras-Champel et al.^63^ | Case series, France |
|  | Atypical thrombosis: CVT, splanchnic and other thrombosis Thrombocytopenia | 1 (F) | 21 | NR | NR | 15 days | NR | NR | NR | Recovered | Gras-Champel et al.^63^ | Case series. France |
|  | Atypical thrombosis: CVT, other thrombosis  DIC | 1 (F) | 69 | NR | NR | 12 days | NR | NR | NR | Died | Gras-Champel et al.^63^ | Case series. France |
|  | Atypical thrombosis: splanchnic and other thrombosis DIC | 1 (F) | 26 | NR | NR | 9 days | NR | NR | NR | Recovered | Gras-Champel et al.^63^ | Case series. France |
|  | Atypical thrombosis: splanchnic and other thrombosis Thrombocytopenia | 1 (M) | 73 | NR | NR | 12 days | NR | NR | NR | Recovered | Gras-Champel et al.^63^ | Case series. France |
|  | Atypical thrombosis: splanchnic and other thrombosis DIC | 1 (F) | 61 | NR | NR | 13 days | NR | NR | NR | Died | Gras-Champel et al.^63^ | Case series. France |
|  | Atypical thrombosis: CVT, splanchnic thrombosis  DIC | 1 (F) | 38 | NR | NR | 8 days | NR | NR | NR | Died | Gras-Champel et al.^63^ | Case series. France |
|  | Atypical thrombosis: other thrombosis  DIC | 1 (F) | 74 | NR | NR | 15 days | NR | NR | NR | Recovered | Gras-Champel et al.^63^ | Case series. France |
|  | Atypical thrombosis: CVT DIC | 1 (M) | 23 | NR | NR | 9 days | NR | NR | NR | Recovered | Gras-Champel et al.^63^ | Case series. France |
|  | Atypical thrombosis: CVT DIC | 1 (F) | 44 | NR | NR | 9 days | NR | NR | NR | Recovered | Gras-Champel et al.^63^ | Case series. France |
|  | Atypical thrombosis: CVT  Thrombocytopenia | 1 (M) | 60 | NR | NR | 11 days | NR | NR | NR | Recovered | Gras-Champel et al.^63^ | Case series. France |
|  | Atypical thrombosis: splanchnic thrombosis  Thrombocytopenia | 1 (M) | 60 | NR | NR | 11 days | NR | NR | NR | Died | Gras-Champel et al.^63^ | Case series. France |
|  | Atypical thrombosis: splanchnic thrombosis  DIC | 1 (M) | 67 | NR | NR | 8 days | NR | NR | NR | Died | Gras-Champel et al.^63^ | Case series. France |
|  | PVT  Venous thrombosis (splenic and superior mesenteric  veins)  Thrombocytopenia | 1 (F) | 36 | None except for history of abdominal pain 17 days prior to vaccine | 1st | Few days | Fever, asthenia, diffuse osteoarticular pain, upper abdominal pain | Platelet count  US Abdominal CT with contrast  Transhepatic portal vein venography | Unfractionated heparin Argatroban  Thrombus aspiration with a penumbra  catheter  Porto-systemic shunt  Accelerated  thrombolysis  rtPA infusion  IVIG  Apixaban (long term) | Recovered | Umbrello et al.^64^ | Case report, Italy |
|  | Arterial thrombosis (suprarenal, left superficial, deep femoral, celiac, and right peroneal arteries)  Thrombocytopenia | 1 (F) | 72 | None | NR | 7 days | Left limb pain and claudication | Unspecified imaging | Unfractionated heparin  Embelectomy Argatroban  High-dose IVIG | Discharge | Bourguignon et al.^65^ | Case series, Canada |
|  | Acute arterial thrombosis in the left leg  DVT (popliteal)  PE  Thrombocytopenia | 1 (M) | 63 | No CVD risk nor thrombosis history | NR | 18 days | Left leg cramping followed by dyspnea and leg became painful and cold | CT angiogram  Lower limb US | Surgical embolectomy LMWH Fondaparinux  IVIG | Amputation | Bourguignon et al.^65^ | Case series, Canada |
|  | CVST (left  transverse sinus extending to the left sigmoid sinus and jugular vein) Thrombocytopenia | 1 (F) | 61 | DM  HTN  DLP  High liver enzymes | 1st | 14 days | left ear pain, headache, dizziness, nausea, tinnitus | CT brain | Enoxaparin | Discharged with NIHSS score of 8 | Abu Esba et al.^28^ | Case series, Saudi Arabia |
|  | CVST Thrombocytopenia | 1 (M) | 40 | No thrombotic risk factors | 1st | 14 days | Severe headache, nausea | CT brain | Enoxaparin  Apixaban | Discharged on apixaban | Abu Esba et al.^28^ | Case series, Saudi Arabia |
|  | CVST Thrombocytopenia | 1 (F) | 47 | No thrombotic risk factors | NR | 7 days | NR | NR | NR | Died | Althaus et al.^66^ | Case series, Germany |
|  | DVT  PE Thrombocytopenia | 1 (M) | 53 | No thrombotic risk factors | NR | 9 days | NR | NR | NR | Discharged | Althaus et al.^66^ | Case series, Germany |
|  | CVST Thrombocytopenia | 1 (F) | 47 | No thrombotic risk factors | NR | 7 days | NR | NR | NR | Discharged | Althaus et al.^66^ | Case series, Germany |
|  | PE Thrombocytopenia | 1 (M) | 32 | No thrombotic risk factors | NR | 20 days | NR | NR | NR | Discharged | Althaus et al.^66^ | Case series, Germany |
|  | CVST Thrombocytopenia | 1 (F) | 36 | No thrombotic risk factors | NR | 17 days | NR | NR | NR | Discharged | Althaus et al.^66^ | Case series, Germany |
|  | CVST Thrombocytopenia | 1 (F) | 29 | No thrombotic risk factors | NR | 7 days | NR | NR | NR | Discharged | Althaus et al.^66^ | Case series, Germany |
|  | CVST (superior sagittal sinus and proximal left transverse sinus) PVT  Venous thrombosis (superior mesenteric vein)  Potential splenic and hepatic infarction  DIC | 1 (F) | 36 | DM | 1st | 14 days | Fever, vomiting, severe headache, focal left-sided convulsions for 5 minutes followed by weakness in the left arm | CT brain  CT venogram  Laboratory data (fibrinogen, hemoglobin, platelets) | Enoxaparin  Antibiotics  Antivirals ICU with intubation, mechanicalventilation, and ionotropic support | Died | Aladdin et al.^67^ | Case report, Saudi Arabia |
|  | Arterial thrombosis (bilateral internal carotids)  PE  CVST (left transverse and sigmoid sinuses)  Jugular vein thrombosis  Venous thrombosis (hepatic and iliac veins)  Border zone infarct Thrombocytopenia | 1 (F) | 37 | NR | NR | 12 days | Diffuse headache, left visual field loss, confusion, left arm weakness | CT angiogram  MRI | IVIG  Methylprednisolone Plasmapheresis  Fondaparinux | Improved | Al-Mayhani et al.^68^ | Case series, United Kingdom |
|  | Venous thrombosis (ophthalmic vein)  Ischemic stroke (MCA and parietal lobe)*  Secondary ITP | 1 (F) | 55 | None | 1st | 10 days | Conjunctival congestion, retro-orbital pain, diplopia | MRI  Platelet suspension immunofluorescence test  Monoclonal antibody-specific immobilisation of platelet antigens assay | IV dexamethasone Therapeutic heparin, Levetiracetam Lacosamide Phenprocoumon | Discharged | Bayas et al.^69^ | Case report, Germany |
|  | TTP | < 5¶ (This number includes TTP and various other arterial events) | 32-55 | NR | 1st | NR | NR | NR | NR | NR | Pottegård et al.^58^ | Cohort study, Denmark and Norway |
|  | CVST (thrombotic occlusion of the straight sinus and a non-occlusive thrombus in the superior sagittal sinus)  Thrombocytopenia | 1 (F) | 36 | None | 1st | 7-17 days | 7 days:  Severe headaches, fever  17 days: Acute somnolence with a right-hand hemiparesis | MRI | Thrombus removal by endovascular rheolysis  Enoxoparin sodium Dabigatran | Recovered | Wolf et al.^70^ | Case series, Germany |
|  | CVST Thrombocytopenia | 4 (NR) | 22-49 | 1 with CND | NR | 7 days (2)  8 days (1)  9 days (1) | NR | CBC | NR (2)  Heparin (2) | Recovered (2)  Died (1) -had CND  NR (1) | Greinacher et al.^71^ (a) | Case series, Germany and Austria |
|  | CVST Thrombocytopenia | 9 (5F/4M) | 25-48 | NR | 1st | 12 days (2)  9 days (2)  14 days (3)  10 days (1)  19 days (1) | NR | NR | NR | Alive (7)  Died (2) | Scully et al.^59^ | Case series, UK |
|  | CVST  PE  Multiple venous thrombosis (splanchnic vein,  right intra-ventricular  iliofemoral vein and IVC) Thrombocytopenia | 1 (NR) | 22-49 | VWD-1 FVL  ACL-Abs | NR | 13 days | NR | CBC | Heparin | Recovered | Greinacher et al.^71^ (a) | Case series, Germany and Austria |
|  | CVST  PVT  PE  Ischemic bowel with infarction Thrombocytopenia | 1 (F) | 30 | NR | 1st | 13 days | NR | NR | NR | Alive | Scully et al.^59^ | Case series, UK |
|  | CVST  Venous thrombosis (splanchnic vein) Thrombocytopenia | 1 (NR) | 22-49 | None | NR | 11 days | NR | CBC | No heparin | Died | Greinacher et al.^71^ (a) | Case series, Germany and Austria |
|  | CVST  Brain, lung, kidneys microvascular thrombosis Thrombocytopenia | 1 (NR) | 22-49 | None | NR | 8 days | NR | CBC | No heparin | Died | Greinacher et al.^71^ (a) | Case series, Germany and Austria |
|  | CVT  Multiple organ thrombi Thrombocytopenia | 1 (NR) | 22-49 | None | NR | 16 days | NR | CBC | No heparin | Died | Greinacher et al.^71^ (a) | Case series, Germany and Austria |
|  | PE  Venous thrombosis (pelvic vein) Thrombocytopenia | 1 (F) | 18-64 | NR | NR | NR | Dyspnea, fatigue | NR | NR | Recovered | Tobaiqy et al.^61^ | Retrospective descriptive study, Saudi Arabia |
|  | PE Thrombocytopenia | 1 (NR) | 22-49 | None | NR | 6 days | NR | CBC | LMWH | Recovered | Greinacher et al.^71^ (a) | Case series, Germany and Austria |
|  | PE Thrombocytopenia | 1 (F) | 49 | NR | 1st | 24 days | NR | NR | NR | Alive | Scully et al.^59^ | Case series, UK |
|  | PVT  Venous thrombosis (left intrahepatic, left hepatic vein, splenic vein, the azygos vein, and the hemiazygos vein) Thrombocytopenia | 1 (M) | 32 | Asthma | 1st | 7 days | Backache | Thoracoabdominal CT  MRI | IVIG Prednisolone  Dalteparin | Recovered | Schultz et al.^72^ | Case series, Norway |
|  | Unspecified thrombosis Thrombocytopenia | 1 (M) | 18-64 | NR | NR | NR | NR | NR | NR | Died | Tobaiqy et al.^61^ | Retrospective descriptive study, Multinational |
|  | Extensive CVST PVT  Thrombophlebitis of the right leg  Thrombocytopenia | 1 (M) | 54 | Congenital limb malformation | NR | 7-21 days | Worsening headache, bruising, unilateral right calf swelling | CT  US  Platelet count | IVIG Anticoagulation with danaparoid and direct oral anticoagulant | Discharged | Ramdeny et al.^73^ | Case report, UK |
|  | Cutaneous thrombosis associated with skin necrosis Microthrombi within blood vessels  Ischemic epidermis and fat necrosis of subcutaneous tissue  Mild thrombocytopenia | 1 (M) | 73 | AFib with ischemic  cardiomyopathy;  takes apixaban among other medications | 1st | 1-14 days | 1 day: fever and headache  3 days: Left shin erythema, rapidly ulcerated blistering  14 days: superficial ulcers with necrotic base and a violaceous edge on the lateral aspect of left shin | Blood tests Punch biopsy | Topical clobetasol propionate Neomycin sulphate Nystatin ointment, Compression bandaging | Recovered | Ramessur et al.^74^ | Case report, UK |
|  | PVT  CVST Thrombocytopenia | 1 (F) | 30 | Migraine  OCP use for years  Heterozygous for the prothrombin mutation (found on workup) | 1st | 8 days | Headache, general malaise, ecchymosis | Platelet count  CT angiogram abdomen  Cerebral CT venography | Tinzaparin Fibrinogen substitute  Fondaparinux  Rivaroxaban | Recovered | Tølbøll Sørensen et al.^75^ | Case report, Denmark |
|  | VITT:  CVST  Thrombocytopenia | 2 (NR) | NR | NR | NR | NR | Altered level of consciousness | NR | IVIG Heparin Corticosteroids Decompressive craniectomy | Died | Geeraerts et al.^76^ | Case series, France |
|  | VITT:  PVT including intrahepatic branches  Venous thrombosis (splenic and superior mesenteric vein)  Thrombocytopenia | 1 (F) | 30 | None | 1st | 8 days | Abdominal and back pain, headache, nausea, vomiting | CT  Duplex US | LMWH | Recovered | Fromme et al.^77^ | Case report, Germany |
|  | VITT:  PVT  Venous thrombosis (hepatic branch vein thrombosis) Thrombocytopenia | 1 (F) | 29 | None | 1st | 7 days | Headach, nausea, leg cramps, bilateral visual disturbance | CT brain and venogram  MR brain/venogram  Doppler US CT Abdomen/pelvis | Apixaban  IVIG  Argatroban | Recovered | Lavin et al.^78^ | Case series, Europe |
|  | Two proximal PEs  Thrombocytopenia | 1 (M) | 38 | Cerebral palsy | 1st | 14 days | Bruising, petechiae | CT pulmonary angiogram | IVIG  Argatroban  Apixaban | Recovered | Lavin et al.^78^ | Case series, Europe |
|  | CVST (right transverse and sigmoid sinuses)  Thrombocytopenia | 1 (F) | 50 | None | 1st | 20 days | Thunderclap headache, nausea, vomiting | CT angiogram  CT venogram | LMWH  Fondaparinux  IVIG  Warfarin | Recovered | Lavin et al.^78^ | Case series, Europe |
|  | Prothrombotic ITP:  CVT (multiple cerebral emboli)  Arterial thrombosis (aortic arch thrombosis)  Cortical infarctions  Thrombocytopenia | 1 (F) | 67 | NR | 1st | 8 days | Headache | Platelet count  Imaging | Argatroban  Dexamethasone  IVIG | Recovered | Tiede et al.^79^ | Case series, USA |
|  | TIA Thrombocytopenia | 1 (F) | 41 | NR | 1st | 5 days | Headach, diplopia | NR | Argatroban | Recovered | Tiede et al.^79^ | Case series, USA |
|  | Prothrombotic ITP:  Venous thrombosis (splanchnic vein thrombosis)  Thrombocytopenia | 1 (F) | 61 | NR | 1st | 9 days | Fatigue | NR | Argatroban  IVIG  Eculizumab | Recovering | Tiede et al.^79^ | Case series, USA |
|  | VITT:  Stroke (proximal left MCA occlusion)  Segmental PE  PVT  Venous thrombosis (splenomesenteric trunk and ileal veins, venous mesenteric ischemia)  Thrombocytopenia | 1 (F) | 26 | NR | NR | NR | Right hemiplegia, aphasia, nausea, headache | CT angiography | Corticosteroids  Plasma exchange  Anticoagulation  Dual thrombo-aspiration to recanalize MCA | Recovering | Garnier et al.^80^ | Case series, Germany |
|  | PE Thrombocytopenia | 1 (F) | 22- 49 | NR | NR | 10 days | NR | NR | NR | Recovering | Greinacher et al.^81^ (b) | Case series, Germany and Austria |
|  | CVST Thrombocytopenia # | 7 (6F/1M) | 22- 49 | 2 autoimmune disease (1 demyelinating disease, 1 APL-Abs) | NR | 4-16 days | NR | NR | NR | Recovering (3)  Died (3)  In hospital (1) | Greinacher et al.^81^ (b) | Case series, Germany and Austria |
|  | VITT:  PVT  Venous thrombosis (splenic veins and mesenteric vein)  Thrombocytopenia | 1 (M) | 44 | Depression on  escitalopram  Heterozygous prothrombin G20210A mutation | 1st | 8 days | Fevers, fatigue, head fogginess, abdominal discomfort,  increased bowel frequency | CT venogram | Fondaparinux)  IVIG  Bivalirudin  Methylprednisone  Laparotomy with bowel resection | Recovered | Hocking et al.^82^ | Case report, Australia |
|  | Ischemic stroke (MCA)  Thrombocytopenia | 2 (1F/1M) | F: 39  M: 21 | NR | 1st | 10 days | NR | NR | NR | Alive | Scully et al.^59^ | Case series, UK |
|  | VITT:  Stroke (MCA thrombosis with malignant MCA infarction)  Arterial thrombosis (Right internal carotid artery)  Thrombocytopenia | 1 (F) | 39 | Migraines  BRCA2 carrier | 1st | 9 days | Left-sided weakness, confusion, headache, nausea | CT w ith angiogram  Platelet count | Aspirin  IVIG  Methylprednisone  Argatroban  Decompressive  hemicraniectomy | In hospital | Jacob et al.^83^ | Case report, UK |
| **Thrombosis and thrombocytopenia with major bleeding (41)** | Arterial thrombosis (carotid artery)  Thrombocytopenia | 1 (F) | 18-64 | NR | NR | NR | Unspecified hemorrhage | NR | NR | Not resolved | Tobaiqy et al.^61^ | Retrospective descriptive study, Multinational |
|  | CVST  PVT  Peripheral PE  Venous thrombosis (splenic and upper mesenteric, splanchnic veins) Arterial thrombosis (infrarenal aorta and both iliac arteries)  Thrombocytopenia | 1 (F) | 49 | NR | 1st | 5 days | Fatigue, myalgia, headache, diffuse GI bleeding | CT | Platelet concentrate  IV Abs  Analgesia  LMWH  Low-dose IV unfractionated heparin  RBCs transfusion Platelet transfusion Prothrombin complex concentrates Recombinant factor VIIa | Died | Greinacher et al.^81^ (b) | Case series, Germany and Austria |
|  | CVT/CVST (SSS thrombosis, vein of Galen)  PE  Arterial thrombosis (aortic arch, thoracic aorta, portal, suprahepatic, right coronary, and basilar arteries)  Thrombocytopenia | 1 (F) | 54 | None | 1st | 2 days | Headache, vomiting, brain and brainstem hemorrhagic infarction with SAH | NR | Enoxaparin  Fondaparinux  Desametasone | Died | Ciccone et al.^34^ | Case series, Italy |
|  | Right MCA stroke with hemorrhagic transformation  CVST (transverse and sigmoid sinuses)  Jugular vein thrombosis  DVT  PE  Arterial thrombosis (internal carotid) Venous thrombosis (hepatic vein)  Thrombocytopenia | 1 (M) | 69 | DM2  HTN  OSA  Prostate cancer Heparin exposure 9 months ago due to transcatheter aortic-valve replacement | NR | 12 days | Headache, confusion, progressive left-sided weakness | NR | Fondaparinux  IVIG  Rivaroxaban  Plasma exchange | Platelets recovered but hemiplegia remained | Bourguignon et al.^65^ | Case series, Canada |
|  | Suspected thrombosis Thrombocytopenia | 1 (NR) | 22-49 | NR | NR | 12 days | ICH | CBC | No heparin | Died | Greinacher et al.^71^ (a) | Case series, Germany and Austria |
|  | CVT/CVST (SSS thrombosis, cortical vein thrombosis)  Severe thrombocytopenia | 1 (M) | 32 | None | 1st | 9 days | Thunderclap headache, left-sided incoordination hemiparesis , ICH (parenchymal and SAH)  Deterioration: reduced GCS, generalised tonic-clonic  Seizures, decerebrate posturing, dilated unreactive pupils | CT head  Platelet count | Deterioration:  Intubation Ventilation Mannitol Hypertonic saline | Died | Mehta et al.^84^ | Case series, UK |
|  | CVST (superior sagittal, left-hand transverse and the sigmoid sinuses)  Thrombocytopenia | 1 (F) | 22 | None | 1st | 4-7 days | Frontally accentuated, self-limited generalized epileptic seizure, pronator drift on left arm, SAH | MRI | Endovascular rheolysis  Levetiracetam  Enoxoparin  Dabigatran | Recovered | Wolf et al.^70^ | Case series, Germany |
|  | CVST (superior sagittal, left-hand transverse and the sigmoid sinuses)  Thrombocytopenia | 1 (F) | 46 | None | 1st | 8-13 days | 8 days:  Headache, mild aphasia  13 days:  Right hemianopia  left occipital lobe hemorrhage | MRI | Partial thrombus removal by endovascular rheolysis  Balloon angioplasty Enoxoparin Danaparoid Dabigatran | Recovered | Wolf et al.^70^ | Case series, Germany |
|  | CVST (left transverse and sigmoid sinuses)  Thrombocytopenia | 1 (M) | 50 | None | 1st | 11 days | Headache, hemiparesis, visual impairment, left intraparenchymal hemorrhage | CT angiogram | Fibrinogen concentrate  Platelets | Died | Castelli et al.^85^ | Case report, France |
|  | CVST (straight, sagittal and transverse sinus)  Bilateral PE  Microthrombi in glomerular arterioles and capillaries Thrombocytopenia | 1 (F) | 48 | NR | NR | 6 days | SAH | NR | NR | Died | Althaus et al.^66^ | Case series, Germany |
|  | Multiple thromboses: Bilateral PE Microthrombi in glomeruli  Thrombocytopenia | 1 (M) | 24 | Heterozygous FVL mutation | NR | 10 days | ICH | NR | NR | Died | Althaus et al.^66^ | Case series, Germany |
|  | Right MCA occlusion with hemorrhagic transformation  PVT Thrombocytopenia | 1 (F) | 35 | NR | NR | 6-11 days | Episodic right temporal and periorbital headache,  left face and arm and leg weakness, right gaze preference, drowsiness | CT angiography | Urgent decompressive hemicraniectomy IVIG Plasmapharesis  Fondaparinux | Brainstem death | Al-Mayhani et al.^68^ | Case series, United Kingdom |
|  | MCA infarct with hemorrhagic transformation Thrombocytopenia | 1 (M) | 43 | NR | NR | 21 days | Dysphasia | CT  MRI | Platelet transfusion IVIG  Fondaparinux | Resolved | Al-Mayhani et al.^68^ | Case series, United Kingdom |
|  | PE  CVST (superior sagittal, left straight and sigmoid sinuses)  Jugular vein thrombosis  Thrombocytopenia | 1 (M) | 50 | None | 1st | 7 days | Headache, massive brain hemorrhage | NR | Enoxaparin  IV mannitol Craniectomy | Died | Ciccone et al.^34^ | Case series, Italy |
|  | CVST (superior sagittal, right straight and sigmoid sinuses)  Jugular vein thrombosis  Venous thrombosis (supra-hepatic veins)  Thrombocytopenia | 1 (F) | 42 | Mutation factor II | 1st | 0 days | Brain hemorrhagic infarction | NR | Enoxaparin  IV mannitol Throbmectomty Craniectomy | In a coma | Ciccone et al.^34^ | Case series, Italy |
|  | CVST (left straight and sigmoid sinuses)  Venous thrombosis (epigastric and periuterin veins thrombosis)  Renal infarction  Thrombocytopenia | 1 (F) | 32 | Thrombocytopenia in infancy with brain hemorrhage  OCP use | 1st | 1 day | Headache, orbital bruising, abdominal pain, fever, cerebellar hemorrhagic infarction | NR | Fondaparinux  Metilprednisolone | Died | Ciccone et al.^34^ | Case series, Italy |
|  | CVST (superior sagittal, right straight and sigmoid sinuses)  PVT  Venous thrombosis (mesenteric veins)  Thrombocytopenia | 1 (F) | 35 | OCP use | 1st | 6 days | Headache, nausea, vomiting, brain hemorrhagic infarction | NR | IV mannitol  IV metilprednisolone  IV fresh plasma Enoxaparin  Plasmapheresis | In a coma | Ciccone et al.^34^ | Case series, Italy |
|  | CVST (left straight, sigmoid sinuses, vein of Galen and internal cerebral veins)  Jugular vein thrombosis  Venous thrombosis (pelvic district)  Thrombocytopenia | 1 (F) | 51 | Heterozygosis for FVL and methyltetrahydrofolate reductase | 1st | 10 days | Headache, vomiting, drowsiness, bilateral deep brain hemorrhagic infarction | NR | IV remifentanil IV noradrenalinVentriculostomy | Died | Ciccone et al.^34^ | Case series, Italy |
|  | CVST (inferior sagittal, left straight and sigmoid sinuses  Jugular vein thrombosis  Thrombocytopenia | 1 (F) | 40 | Anamnestic spontaneous abortion | 1st | 5 days | Headache, brain hemorrhagic infarction | NR | Fondaparinux | Aphasia and right hemiparesis | Ciccone et al.^34^ | Case series, Italy |
|  | Jugular venous thrombosis  PE  PVT  Venous thrombosis  (IVC) Thrombocytopenia | 1 (F) | 55 | None | 1st | 6 days | Headache, fever, cerebellar hemorrhagic infarction | NR | Fondaparinux  IV mannitol IV metilprednisolone  Craniectomy | In a coma | Ciccone et al.^34^ | Case series, Italy |
|  | CVST (inferior sagittal, straight, right transverse and right sigmoid sinuses and deep and superficial cerebral veins and vein of Galen)  Thrombocytopenia | 1 (F) | 39 | None | 1st | 8 days | Headache, abdominal pain, right cerebellar hemorrhage | Cerebral CT with venography | Dalteparin Prednisolone  IVIG | Recovered | Schultz et al.^72^ | Case series, Norway |
|  | CVST (cortical veins, superior sagittal sinus, both transverse sinuses, and left sigmoid sinus)  Thrombocytopenia | 1 (F) | 54 | HTN | 1st | 7 days | Hemiparesis on the left side of body, headache, right frontal hemorrhage | CT of the head with venography | Platelet transfusion  Methylprednisolone  IVIG  Unfractioned heparin  Endovascular intervention with thrombectomy  Decompressive hemicraniectomy | Died | Schultz et al.^72^ | Case series, Norway |
|  | CVST Thrombocytopenia | 1 (F) | 22 | NR | 1st | 10 days | ICH | NR | NR | Died | Scully et al.^59^ | Case series, UK |
|  | CVST (left transverse and sigmoid sinuses)  Thrombocytopenia | 1 (M) | 50 | None | 1st | 7 days | Worsening headache, left intraparenchymal hemorrhage | CT with angiography | ICU urgent neurosurgery intervention | Died | Franchini et al.^86^ | Case report, Italy |
|  | PE  CVT  Arterial thrombosis (peripheral artery)  Thrombocytopenia | 1 (F) | 18-64 | NR | NR | NR | ICH | NR | NR | Not resolved | Tobaiqy et al.^61^ | Retrospective descriptive study, Multinational |
|  | CVST  PVT  PE  Arterial thrombosis (aortoilliac)  Venous thrombosis (splanchnic vein)  Thrombocytopenia | 1 (NR) | 22-49 | None | 1st | 5 days | Chills, fever, nausea, epigastric discomfort, diffuse GI bleeding | CBC | Heparin | Died | Greinacher et al.^71^ (a) | Case series, Germany and Austria |
|  | CVST  Jugular vein thrombosis Thrombocytopenia | 1 (F) | 49 | NR | 1st | 15 days | SAH | NR | NR | Alive | Scully et al.^59^ | Case series, UK |
|  | CVST  Multiple organ thrombosis (lungs and intestines) Thrombocytopenia | 1 (F) | 52 | NR | 1st | 10 days | ICH | NR | NR | Died | Scully et al.^59^ | Case series, UK |
|  | CVST (left transverse and sigmoid sinuses) Thrombocytopenia | 1 (F) | 37 | Pollen allergy | 1st | 7 days | Fever, persistent headache, visual disturbances, cerebellar hemorrhage | CT | Dalteparin  Platelet transfusions Decompressive craniectomy | Died | Schultz et al.^72^ | Case series, Norway |
|  | CVST (transverse and sigmoid sinuses) Thrombocytopenia | 1 (F) | 42 | Pollen allergy | 1st | 7 days | Headache,  reduced consciousness/drowsiness, hemorrhagic infarction in the left hemisphere | CT venography | Dalteparin  Hemicraniectomy  Platelet transfusions  Methylprednisolone  IVIG | Died | Schultz et al.^72^ | Case series, Norway |
|  | CVST (superior sagittal sinus thrombosis with extension into the cortical veins)  Thrombocytopenia | 1 (M) | 25 | PSC Migraines | 1st | 6 days | Meningitic, headache, photophobia, vomiting, petechial rash, gum bleeding, left hemiparesis and hemisensory loss, lobar hemorrhage and SAH  Deterioration: Seizures, agitation, decerebrate posturing, reduced GCS | CT head  Platelet count | Unfractioned heparin  Dexamethasone  IVIG  Platelet transfusion  IV levetiracetam  Deterioration: sedation and intubation | Died | Mehta et al.^84^ | Case series, UK |
|  | DVT Thrombocytopenia | 1 (M) | 66 | NR | 1st | 12 days | Adrenal hemorrhage | NR | NR | Alive | Scully et al.^59^ | Case series, UK |
|  | PVT  Arterial thrombosis (aortic) Thrombocytopenia | 1 (F) | 55 | None | 1st | 6 days | ICH | NR | NR | Died | Scully et al.^59^ | Case series, UK |
|  | Unspecified thrombosis Thrombocytopenia | 1 (F) | 71 | NR | 1st | 14 days | Hemorrhagic symptoms | NR | NR | Alive | Scully et al.^59^ | Case series, UK |
|  | CVST (sigmoid and superior sagittal sinuses)  Jugular vein thrombosis  PE  Thrombocytopenia | 1 (F) | 69 | HTN | 1st | 11 days | Headache, behavioral changes, unconsciousness, frontal hemorrhage | CT  MRI | Intubation Transfer to ICU Supportive care | Died | Jamme et al.^87^ | Case report, France |
|  | Prothrombotic ITP:  CVST (left transverse and sigmoid sinuses)  Thrombotic microangiopathy | 1 (F) | 63 | NR | 1st | 11 days | Headache, somnolence,dysphasia, right sided hemiparesis, arterial HTN, left temporal hemorrhage | Platelet count  Imaging | Unfractionated heparin  Dexamethasone  Eculizumab | Recovering | Tiede et al.^79^ | Case series, USA |
|  | Prothrombotic ITP:  MCA infarct with hemorrhagic transformation  Arterial thrombosis (right internal carotid and popliteal artery thrombosis) | 1 (F) | 61 | NR | 1st | 9 days | Headache, dysarthria, left-sided hemiplegia, conjugated gaze palsy | NR | Argatroban  Dexamethasone  IVIG | Recovering | Tiede et al.^79^ | Case series, USA |
|  | Ischemic stroke (MCA infract)  Arterial thrombosis (right internal carotid artery)  Thrombocytopenia | 1 (F) | 60 | HTN  Hashimoto’s thyroiditis | 1st | 7 days | Headache, weakness, bilateral adrenal hemorrhages | MR Angiogram | Hydrocortison  Cefuroxime  Platelet concentrate  Hemicraniectomy | Died | Blauenfeldt et al.^88^ | Case series, Denmark |
|  | CVST Thrombocytopenia | 1 (F) | 47 | None | 1st | 12 days | Progressive headaches, ICH | Head CT including venogram | IVIG  Corticosteroids  Platelets  Surgical decompression  Argatroban | Died | Gessler et al.^89^ | Case series, Germany |
|  | CVST Thrombocytopenia | 1 (F) | 50 | None | 1st | 7 days | Progressive headaches, ICH | Head CT including venogram | IVIG  Corticosteroids  Platelets  Surgical decompression  Argatroban | Died | Gessler et al.^89^ | Case series. Germany |
|  | **VITT:**  CVST (right transverse sinus) | 1 (M) | 27 | None | 1st | 2 days | Intermittent headaches, eye floaters, vomiting, worsening headache with homonymous hemianopsia, parenchymal  hemorrhage in the right parietal lobe | D-dimer  Platelet count Fibrinogen CT venogram | IVIG  Dabigatan  Idarucizumab  Prednisolone  Emergency decompressive craniotomy | Died | Suresh et al.^90^ | Case report, UK |
| **Thrombosis with suspected thrombocytopenia (45) @** | CVST % | 37 (NR) | 20-89 | NR | NR | NR | NR | NR | NR | Died (8) | Schulz et al.^35^ | Case series, Germany |
|  | Ischemic Stroke %% | 8 (NR) | 31-82 | NR | 1st | NR | NR | NR | NR | Died (2) | Schulz et al.^35^ | Case series, Germany |
| **Thrombosis with hemorrhage (2)** | CVST (left straight and sigmoid sinuses thrombosis), jugular vein thrombosis and ICH (brain hemorrhagic infarct) | 1 (F) | 49 | Migraine with aura | 1st | 11 days | Headache | NR | Enoxaparin  IV mannitol | Significant disability on day 20 | Ciccone et al.^34^ | Case series, Italy |
|  | Unspecified thrombosis  Uterine hemorrhage | 1 (F) | 18-64 | NR | NR | NR | NR | NR | NR | Recovered | Tobaiqy et al.^61^ | Retrospective descriptive study, Multinational |
| **Thrombocytopenia with no to minor bleeding (>13)** | DIC | 1 (F) | 52 | Hepatitis B Headache  Left breast cancer treated with bilateral mastectomy  Left ovarian cyst treated with oophorectomy and salpingectomy | 1st | 14 days | Throbbing headache, photophobia, nausea, chills, fever, muscle, joint pain, inability to walk due to severe asthenia, large ecchymoses of the left buttock | Laboratory results | Enoxaparin  IV dexamethasone | Resolved | Casucci et al.^91^ | Case report, Italy |
|  | Thrombocytopenia  Hypofibrinogenemia | 1 (F) | 35 | Migraines | NR | 14 days | Following vaccination: general myalgia, extreme fatigue  10 days: bruising, petechiae  14 days: petechiae, persistent bruising, headache, slightly different from usual migraine | Coagulation screen (platelets, fibrinogen) D-Dimer  Peripheral blood smear MR cerebral venogram | Anticoagulated with apixaban | Recovered | Ryan et al.^92^ | Case report, Ireland |
|  | Thrombocytopenia | 8 (NR) | 21–69 | NR | 1st | 20 (11-35) days | Fever, headache, fatigue, malaise, muscle/joint ache, skin bleeding/bruising | Platelet count  Antibodies to Platelet Factor 4 by ELISA | NR | NR | Sørvoll et al.^93^ | Observational study, Norway |
|  | ITP | 1 (M) | 28 | None | 1st | 22 days | Oral bleeding, petechia over the trunk, arms and legs | Platelet count | Dexamethasone | Recovered | Candelli et al.^94^ | Case report, Italy |
|  | Thrombocytopenia | 17 ¶ | 32-55 | NR | 1st | NR | NR | NR | NR | NR | Pottegård et al.^58^ | Cohort study, Denmark and Norway |
|  | Thrombocytopenia | 1 (F) | 35 | NR | 1st | 10 days | Petechiae, bruising, headache | MR venogram (brain) | Apixaban | Recovered | Lavin et al.^78^ | Case series, Europe |
|  | ITP | 1 (F) | 50 | None | 1st | 3-10 days | Severe back pain, headache | Platelet count  D-dimer  Fibrinogen  Anti-PF4 antibodies  Contrast-enhanced MRI abdomen and pelvis  CT pulmonary angiography | IVIG Dexamethasone  IV argatroban Dabigatran | Recovering | Guetl et al.^95^ | Case report, Austria |
| **Thrombocytopenia with major bleeding (1)** | Vaccine induced prothrombotic immune thrombocytopenia (VIPIT) | 1 (F) | 62 | Hypothyroidism | 1st | 1 day | Aching joints, fever, headache, dizziness, excess bleeding, petechiae, hematoma, gum bleeding | CT scan  Anti-Platelet Factor 4  Heparin IgG immunoassay | Low dose fibrinogen concentrate Short acting danaparoid sulfate  IVIG  Prednisone Apixaban | Recovered | Thaler et al.^96^ | Case report, Austria |
| **Hemorrhage (NR)** | ICH | <5 ¶ | 32-55 | NR | 1st | NR | NR | NR | NR | NR | Pottegård et al.^58^ | Cohort study, Denmark and Norway |
|  | ICH | 8 ¶ | 32-55 | NR | 1st | NR | NR | NR | NR | NR | Pottegård et al.^58^ | Cohort study, Denmark and Norway |
|  | Bleeding (includes Anemia from bleeding, bleeding from respiratory tract, hematuria, intestinal bleeding and other unspecified causes of bleeding) | 74 ¶ | 32-55 | NR | 1st | NR | NR | NR | NR | NR | Pottegård et al.^58^ | Cohort study, Denmark and Norway |
| **Hemorrhage with suspected thrombocytopenia (4)** | ICH %%% | 4 (3F/1M) | 24-53 | None | 1st | NR | NR | NR | NR | Died (1) | Schulz et al.^35^ | Case series, Germany |

*Patient developed focal seizures 8 days after stroke.

@The case series does not separate the age-range, symptoms, which vaccine, and whether it was first or second dose for each patient; instead they were all grouped together.. More information may be found in Supplementary Table 1 of the article.

¶ The number reported represents the number of events and the number of individuals. However, there is an overlap between the groups in each event. Therefore, the total number of events does not reflect the number of cases.

**Abs**: Antibodies; **ACL**: Anti-cardiolipin; **ACS**: Acute Coronary Syndrome; **AFib**: Atrial Fibrillation; **APL-Abs**: Antiphospholipid Antibodies; **BRCA2**: Breast Cancer Gene 2; **CAD**: Coronary Artery Disease; **CBC**: Complete Blood Count; **CK-MB**: Creatine Kinase-Myocardial Band; **CND**: Chronic Neurological Disorder; **CT**: Computed Tomography; **CVD**: Cardiovascular Disease; **CVST**: Cerebral Venous Sinus Thrombosis; **CVT**: Cerebral Venous Thrombosis; **DIC**: Disseminated Intravascular Coagulation; **DLP**: Dyslipidemia; **DM**: Diabetes Mellitus; **DM2**: Type 2 Diabetes Mellitus; **DVT**: Deep Venous Thrombosis; **ECG**: Electrocardiogram; **ELISA**: Enzyme-Linked Immunoassay; **F**: Female; **FVL**: Factor V Leiden; **GI**: Gastrointestinal; **GSC**: Glasgow Coma Scale; **HTN**: Hypertension; **ICH**: Intracerebral Hemorrhage; **ICU**: Intensive Care Unit; **ITP**: Immune Thrombocytopenic Purpura; **IV**: Intravenous; **IVC**: Inferior Vena Cava; **IVIG**: Intravenous Immunoglobulin; **LAD**: Left Anterior Descending Artery; **LMWH**: Low Molecular Weight Heparin; **M**: Male; **MCA**: Middle Cerebral Artery; **MI**: Myocardial Infarction; **MR**: Magnetic Resonance; **MRI**: Magnetic Resonance Imaging; **NIHSS**: National Institutes of Health Stroke Scale; **NR**: Not Reported; **OCP**: Oral Contraceptive Pills; **OSA**: Obstructive Sleep Apnea; **PE**: Pulmonary Embolism; **PSC**: Primary Sclerosing Cholangitis; **PVT**: Portal Vein Thrombosis; **RBC**: Red Blood Cell; **rtPA**: Recombinant Tissue Plasminogen Activator; **SAH**: Subarachnoid Hemorrhage; **SSS**: Superior Sagittal Sinus; **STEMI**: ST Elevation Myocardial Infarction; **TIA**: Transient Ischemic Attack; **TTP**: Thrombotic Thrombocytopenic Purpura; **US**: Ultrasound; **VIPIT**: Vaccine Induced Prothrombotic Immune Thrombocytopenia; **VITT**: Vaccine Induced Immune Thrombotic Thrombocytopenia; **VWD-1**: Von Willebrand’s Disease Type 1
