# Supplementary material for: Cardiovascular and haematological events post COVID‐19 vaccination: A systematic review
Source: J Cell Mol Med. 2021 Dec 29;26(3):636–53. doi: 10.1111/jcmm.17137 (PMC8817142; doi:10.1111/jcmm.17137)
Supplement: Supplementary file 6 — Table S4 [file JCMM-26-636-s006.docx]

**Supplementary Table 4: Types of cardiovascular abnormalities in 21 adult Johnson & Johnson’s vaccinated patients who developed cardiovascular disease in the included case reports/series.**

| **Type of Event** | **Event** | **N (Sex)** | **Age** | **Comorbidities** | **Which dose** | **Onset of symptoms** | **Signs and Symptoms** | **Diagnostic Method** | **Treatment** | **Outcome** | **References** | **Study and Country** |
| --- | --- | --- | --- | --- | --- | --- | --- | --- | --- | --- | --- | --- |
| **Cardiac only (1)** | Acute myocarditis-like illness | 1 (M) | 28 | NR | Single dose vaccine | 5 days | Non-pleuritic non-exertional chest pain | Troponin level  ECG  Echocardiogram  CXR  cMRI  Coronary angiography | Beta Blocker ACE-I  Aspirin Clopidogrel | Recovered | Rosner et al.^10^ | Case series, USA |
| **Thrombocytopenia with no to minor bleeding (1)** | ITP flare | 1 (M) | 60 | ITP  Splenectomy | Single dose vaccine | 5 days | Petechiae, ecchymoses, epistaxis, oral blood blisters | Platelet count | Prednisone  IVIG  Romiplostim  Rituximab | Improved | Kuter et al.^42^ | Case series, USA |
| **Thrombosis and thrombocytopenia with no to minor bleeding (10)** | CVST (right transverse and sigmoid sinuses) PE PVT Thrombocytopenia | 1 (F) | 18-39 | NR* | Single dose vaccine | 8 days | Headaches, nausea,  myalgia, chills, fever | CT angiography  US abdomen MRI  Platelet count | Non-heparin anticoagulation  IVIG Systemic corticosteroids  platelet transfusion | NR* | See et al.^97^ | Case series, USA |
|  | CVST (right transverse sinuses) Jugular vein thrombosis Bilateral DVT Thrombocytopenia | 1 (F) | 18-39 | NR* | Single dose vaccine | 6 days | Chills, dyspnea, fever,  headache | Platelet count  MRI  US | Non-heparin anticoagulation  IVIG Systemic corticosteroids  Platelet transfusion | NR* | See et al.^97^ | Case series, USA |
|  | CVST (superior sagittal,  transverse,  straight and possible  sigmoid sinuses)  Jugular vein thrombosis  Thrombocytopenia | 1 (F) | 18-39 | NR* | Single dose vaccine | 15 days | Headache, neck pain,  nausea, vomiting,  photophobia | NR | Non-heparin anticoagulation  IVIG Systemic corticosteroids  Platelet transfusion | NR* | See et al.^97^ | Case series, USA |
|  | CVST (tight transverse and sigmoid sinuses)  Jugular vein thrombosis  DVT  PE  Thrombocytopenia | 1 (F) | 18-39 | NR* | Single dose vaccine | 10 days | Headache | NR | Non-heparin anticoagulation  IVIG Systemic corticosteroids  Platelet transfusion | NR* | See et al.^97^ | Case series, USA |
|  | CVST (left transverse  and sigmoid sinuses)  Jugular vein thrombosis  PE  Thrombocytopenia | 1 (F) | ≥40 | NR* | Single dose vaccine | 6 days | Headache, petechial rash,  neck pain, photophobia,  body aches | NR | Non-heparin anticoagulation  IVIG Systemic corticosteroids  platelet transfusion | NR* | See et al.^97^ | Case series, USA |
|  | TTP | 1 (F) | 62 | HTN  HLD HypothyroidismGERD | Single dose vaccine | 37 days | Altered mental status | Platelets Haptoglobin ADAMTS13 | Hemodialysis  Packed RBCs  Plasma exchange Methylprednisolone | NR | Yocum et al.^98^ | Case report, USA |
|  | **TTS**  Arterial thrombosis (Right carotid artery)  Venous thrombosis (left brachial and right femoral veins)  Thrombocytopenia | 1 (F) | 30-39 | NR | Single dose vaccine | 10 days | Headache, left-sided paresis | NR | NR | Alive | Shay et al.^99^ | Case series, USA |
|  | **TTS**  DVT  Arterial thrombosis (right femoral artery, left and right iliac arteries) Thrombocytopenia | 1 (F) | 50-59 | NR | Single dose vaccine | 11 days | Left leg swelling, bruising | NR | NR | Alive | Shay et al.^99^ | Case series, USA |
|  | **TTS**  PVT  PE  Arterial thrombosis (superior mesenteric and splenic arteries) Thrombocytopenia | 1 (F) | 30-39 | NR | Single dose vaccine | 6 days | Nausea, vomiting, SOB, altered mental status | NR | NR | Alive | Shay et al.^99^ | Case series, USA |
|  | CVST  PE  Thrombocytopenia | 1 (F) | 40 | Migraines  Obesity | Single dose vaccine | 6 days | Sudden headache, body aches, fever, chills | Platelet and D-dimer level  CT head  Platelet factor-4 ELISA (+) | Nonheparin anticoagulant  Prednisone IVIG  Rivaroxaban | Recovered | Costello et al.^100^ | Case Report, USA |
| **Thrombosis and thrombocytopenia with major bleeding (9)** | **VITT**  CVST (right transverse and straight sinuses)  Venous thrombosis (splanchnic, right hepatic and splenic veins)  Thrombocytopenia | 1 (F) | 48 | None | Single dose vaccine | 14 days | 3 day history of malaise, abdominal pain, headache, hemorrhagic stroke | Peripheral-blood smear  Blood test (fibrinogen, aPTT, D-dimer)  CT abdomen and pelvis  Head CT  MRI and magnetic resonance venography of the brain  Repeat CT angiography | Unfractionated heparin (was then switched to argatroban)  IVIG | In-hospital | Muir et al.^101^ | Case report, USA |
|  | CVST  Thrombocytopenia | 1 (F) | 44 | None | Single dose vaccine | 10 days | Progressive headaches, ICH | Head CT with venogram | IVIG  Corticosteroids  Platelets  Surgical decompression  Argatroban | Died | Gessler et al.^89^ | Case series, Germany |
|  | CVST (right transverse  and sigmoid sinuses)  Thrombocytopenia | 1 (F) | ≥40 | NR* | Single dose vaccine | 6 days | Headache, lethargy then severe headache,  left-sided weakness,  dry heaving, large right temporoparietal hemorrhage | CT angiogram  Platelet count | NR | NR* | See et al.^97^ | Case series, USA |
|  | CVST (confluence of sinuses, straight, left transverse and left sigmoid sinuses) Jugular vein thrombosis Thrombocytopenia | 1 (F) | 18-39 | NR* | Single dose vaccine | 9 days | Headache then aphasia, left temporal lobe hemorrhage | CT head  Platelet count CT venogram | NR | NR* | See et al.^97^ | Case series, USA |
|  | CVST (SSS thrombosis) Thrombocytopenia | 1 (F) | 18-39 | NR* | Single dose vaccine | 8 days | Headaches, fever,  vomiting then  left arm weakness, gaze  deviation, left neglect,  seizure, changes in speech, right frontal lobe hemorrhage, right SAH | CT angiogram  MRI  Venogram  Platelet count | NR | NR* | See et al.^97^ | Case series, USA |
|  | CVST (straight and right transverse sinuses) PVT DVT (right posterior tibial and peroneal veins) Venous thrombosis (splenic, right hepatic and distal superior mesenteric veins) Thrombocytopenia | 1 (F) | ≥40 | NR* | Single dose vaccine | 13 days | Back pain, bruising,  abdominal pain then headache, mild retroperitoneal, intraperitoneal, and pelvic hemorrhage, right occipital lobe hemorrhage | CT  Platelet count | NR | NR* | See et al.^97^ | Case series, USA |
|  | CVST (SSS and bilateral cortical veins thrombosis) Thrombocytopenia | 1 (F) | ≥40 | NR* | Single dose vaccine | 7 days | Headache, cognitive  fogginess, right arm  weakness later developed  aphasia, seizure, bilateral frontal lobes hemorrhage | NR | NR | NR* | See et al.^97^ | Case series, USA |
|  | CVST (superior sagittal, right transverse and sigmoid sinuses) Thrombocytopenia | 1 (F) | 18-39 | NR* | Single dose vaccine | 7 days | Headache, nausea,  vomiting, photophobia then developed  loss of consciousness,  seizure, right temporal lobe and left cerebellar hemisphere hemorrhage, SAH | NR | NR | NR* | See et al.^97^ | Case series, USA |
|  | CVST (Torcula, bilateral transverse and right sigmoid sinuses) Bilateral jugular vein thrombosis Thrombocytopenia | 1 (F) | 18-39 | NR* | Single dose vaccine | 11 days | Headache, blurry vision then neck stiffness,  Vomiting, right posterior temporal lobe hemorrhage | NR | NR | NR* | See et al.^97^ | Case series, USA |

*****Among all this study’s patients, 6 had obesity, 1 OCP use, 1 hypothyroidism and 1 reported previous COVID-19 infection. In addition, 3 died, 5 were in-hospital, 4 were discharged and of the 9 alive, 6 recovered.

**ACE-I**: Angiotensin-Converting Enzyme Inhibitors; **ADAMTS13**: A Disintegrin and Metalloproteinase with a Thrombospondin type 1 motif, member 13; **aPTT**: Activated Partial Thromboplastin Time; **cMRI**: Cardiac Magnetic Resonance Imaging; **CT**: Computed Tomography; **CVST**: Cerebral Venous Sinus Thrombosis; **CXR**: Chest X-Ray; **DVT**: Deep Venous Thrombosis; **ECG**: Electrocardiogram; **ELISA**: Enzyme-Linked Immunoassay; **F**: Female; **GERD**: Gastroesophageal Reflux Disease; **HLD**: Hyperlipidemia; **HTN**: Hypertension; **ICH**: Intracerebral Hemorrhage; **ITP**: Immune Thrombocytopenic Purpura; **IVIG**: Intravenous Immune Globulin; **M**: Male; **MRI**: Magnetic Resonance Imaging; **NR**: Not Reported; **OCP**: Oral Contraceptive Pills; **PE**: Pulmonary Embolism; **PVT**: Portal Vein Thrombosis; **RBC**: Red Blood Cell; **SAH**: Subarachnoid Hemorrhage; **SSS**: Superior Sagittal Sinus; **TTP**: Thrombotic Thrombocytopenic Purpura; **TTS**: Thrombotic Thrombocytopenic Syndrome; **US**: Ultrasound; **VITT**: Vaccine Induced Immune Thrombotic Thrombocytopenia
