# Supplementary material for: Cardiovascular and haematological events post COVID‐19 vaccination: A systematic review
Source: J Cell Mol Med. 2021 Dec 29;26(3):636–53. doi: 10.1111/jcmm.17137 (PMC8817142; doi:10.1111/jcmm.17137)
Supplement: Supplementary file 7 — Table S5 [file JCMM-26-636-s003.docx]

**Supplementary Table 5: Types of cardiovascular abnormalities in 2 adult CoronoVac vaccinated patients who developed cardiovascular disease in the included case reports/series.**

| **Type of Event** | **Event** | **N (Sex)** | **Age** | **Comorbidities** | **Which dose** | **Onset of symptoms** | **Signs and Symptoms** | **Diagnostic Method** | **Treatment** | **Outcome** | **References** | **Study and Country** |
| --- | --- | --- | --- | --- | --- | --- | --- | --- | --- | --- | --- | --- |
| **Cardiac only (1)** | Kounis Syndrome Type I variant | 1 (F) | 41 | None | 1st | 15 minutes | Flushing, palpitations, lip and tongue swelling, SOB, chest pain | ECG  Transthoracic echocardiogram  Troponin level CK-MB level  Coronary angiography  Ventriculography | IV pheniramine maleate  Dexamethasone  Oxygen  Salbutamol  IM Epinephrine  Aspirin  Antihistamines  Diltiazem  Corticosteroids | Recovered | Özdemir et al.^102^ | Case report, Turkey |
| **Thrombocytopenia with no to minor bleeding (1)** | Hemophagocytic Lymphohistiocytosis | 1 (F) | 43 | None | 1st | “Shortly after” | Malaise, vomiting, fever | CBC with coagulation  Hepatic and renal function tests  Fasting lipid testing  Ferritin  EBV serology (+)  Bone marrow testing | Antibiotics NSAIDs  Dexamethasone acetat | Recovering | Tang and Hu^103^ | Case report, China |

**CBC**: Complete Blood Count; **CK-MB**: Creatine Kinase-Myocardial Band; **EBV**: Epstein-Barr Virus; **ECG**: Electrocardiogram; **F**: Female; **IM**: Intramuscular; **IV**: Intravenous; **M**: Male; **NSAIDs**: Non-Steroidal Anti-Inflammatory Drugs; **SOB**: Shortness of Breath.
